# Comparative Performance of Four Single Extreme Outlier Discordancy Tests from Monte Carlo Simulations

**DOI:** 10.1155/2014/746451

**Published:** 2014-03-11

**Authors:** Surendra P. Verma, Lorena Díaz-González, Mauricio Rosales-Rivera, Alfredo Quiroz-Ruiz

**Affiliations:** ^1^Departamento de Sistemas Energéticos, Instituto de Energías Renovables, Universidad Nacional Autónoma de México, 62580 Temixco, MOR, Mexico; ^2^Facultad de Ciencias, Universidad Autónoma del Estado de Morelos, 62209 Cuernavaca, MOR, Mexico; ^3^Posgrado en Ciencias, Facultad de Ciencias, Universidad Autónoma del Estado de Morelos, 62209 Cuernavaca, MOR, Mexico; ^4^Departamento de Computación, Instituto de Energías Renovables, Universidad Nacional Autónoma de México, 62580 Temixco, MOR, Mexico

## Abstract

Using highly precise and accurate Monte Carlo simulations of 20,000,000 replications and 102 independent simulation experiments with extremely low simulation errors and total uncertainties, we evaluated the performance of four single outlier discordancy tests (Grubbs test N2, Dixon test N8, skewness test N14, and kurtosis test N15) for normal samples of sizes 5 to 20. Statistical contaminations of a single observation resulting from parameters called *δ* from ±0.1 up to ±20 for modeling the slippage of central tendency or *ε* from ±1.1 up to ±200 for slippage of dispersion, as well as no contamination (*δ* = 0 and *ε* = ±1), were simulated. Because of the use of precise and accurate random and normally distributed simulated data, very large replications, and a large number of independent experiments, this paper presents a novel approach for precise and accurate estimations of power functions of four popular discordancy tests and, therefore, should not be considered as a simple simulation exercise unrelated to probability and statistics. From both criteria of the Power of Test proposed by Hayes and Kinsella and the Test Performance Criterion of Barnett and Lewis, Dixon test N8 performs less well than the other three tests. The overall performance of these four tests could be summarized as N2≅N15 > N14 > N8.

## 1. Introduction

As summarized by Barnett and Lewis [1], a large number of discordancy tests are available for determining an outlier as an extreme (i.e., legitimate) or a discordant (i.e., contaminant) observation in normal samples at a given confidence or significance level. These discordancy tests are likely to be characterized by different power or performance. Numerous researchers [1–6] have commented on the properties of these tests under the slippage of location or central tendency and slippage of scale or dispersion by one or more observations, but very few studies have been reported on the use of Monte Carlo simulation for precise and accurate performance measures of these tests. Relatively more recently using Monte Carlo simulation of *M* = 100,000 replications or runs, Hayes and Kinsella [7] evaluated the performance criteria of two discordancy tests (Grubbs single outlier test N2 and Grubbs multiple outlier test N4k2; the nomenclature is after Barnett and Lewis [1]) and discussed their spurious and nonspurious components of type II error and power function. However, four single extreme outlier type discordancy tests, also called two-sided discordancy tests by Barnett and Lewis [1], are available, which are Grubbs type N2, Dixon type N8, skewness N14, and kurtosis N15. Their relative performance measures should be useful for choosing among the different tests for specific applications.

Monte Carlo simulation methods have been extensively used in numerous simulation studies [8–18]. Some of the relatively recent papers are by Efstathiou [12], Gottardo et al. [13], Khedhiri and Montasser [14], P. A. Patel and J. S. Patel [15], Noughabi and Arghami [16], Krishnamoorthy and Lian [17], and Verma [18]. For example, Noughabi and Arghami [16] compared seven normality tests (Kolmogorov-Smirnov, Anderson-Darling, Kuiper, Jarque-Bera, Cramer von Mises, Shapiro-Wilk, and Vasicek) for sample sizes of 10, 20, 30, and 50 and under different circumstances recommended the use of Jarque-Bera, Anderson-Darling, Shapiro-Wilk, and Vasicek tests.

We used Monte Carlo simulations to evaluate comparative efficiency of four extreme outlier type discordancy tests (N2, N8, N14, and N15, the nomenclature after Barnett and Lewis [1]) for sample sizes of 5 to 20. Our approach to the statistical problem of test performance is novel because, instead of using commercial or freely available software, we programmed and generated extremely precise and accurate random numbers and normally distributed data, used very large replications of 20,000,000, performed 102 independent experiments, and reduced the simulation errors to such an extent that the differences in test performance are far greater than the total uncertainties expressed as 99% confidence intervals of the mean. This is an approach hitherto practiced by none (see, e.g., [8–18]) except by our group [19–23]. This work, therefore, supersedes the approximate simulation results of test performance reported by the statisticians Hayes and Kinsella [7].

## 2. Discordancy Tests

For a data array *x*_1_, *x*_2_, *x*_3_,…, *x*_*n*−2_, *x*_*n*−1_, *x*_*n*_ or an ordered array *x*_(1)_, *x*_(2)_, *x*_(3)_,…, *x*_(*n*−2)_, *x*_(*n*−1)_, *x*_(*n*)_ of *n* observations, with mean $\overset{-}{x}$ and standard deviation *s*, four test statistics were objectively evaluated in this work. For a statistically contaminated sample of size of 5 to 20, *n* − 1 observations of this data array were obtained from a normal distribution *N*(0,1) and the remaining observation was taken from a central tendency shifted distribution *N*(0 + *δ*, 1) or dispersion shifted distribution *N*(0,1 × *ε*), where the contaminant parameters *δ* for modeling slippage of central tendency and *ε* for slippage of dispersion can be either positive or negative. For an uncontaminated sample, the simulations were done for *δ* = 0 and *ε* = ±1. In order to achieve an unbiased comparison, the application of the tests was always forced to the upper outlier *x*_(*n*)_ for positive values of *δ* or *ε* and to the lower outlier *x*_(1)_ for negative values of *δ* or *ε*.

Thus, the first test was the Grubbs test N2 [24] for an extreme outlier *x*_(*n*)_ or *x*_(1)_, for which the test statistic is as follows:

(1) $$\begin{array}{c} \text{TN}2=\{\begin{array}{cc} \frac{x_{\left(n\right)}-\overset{-}{x}}{s},\;x_{\left(n\right)}\;\;\text{tested}\;\;\text{if}\;\;\delta >0\;\;\text{or}\;\;\varepsilon >1 & \\ \frac{\overset{-}{x}-x_{\left(1\right)}}{s},\;x_{\left(1\right)}\;\;\text{tested}\;\;\text{if}\;\;\delta <0\;\;\text{or}\;\;\varepsilon <1 & \\ \mathrm{Max}\left(\frac{x_{\left(n\right)}-\overset{-}{x}}{s},\frac{\overset{-}{x}-x_{\left(1\right)}}{s}\right), & \\ \;\;\;x_{\left(n\right)}\;\;\text{or}\;\;x_{\left(1\right)}\;\;\text{tested}\;\;\text{if}\;\;\delta =0\;\text{or}\;\;\varepsilon =1. & \end{array} \end{array}$$

The second test was the Dixon test N8 [2] as follows:

(2) $$\begin{array}{c} \text{TN}8=\{\begin{array}{cc} \frac{x_{\left(n\right)}-x_{\left(n-1\right)}}{x_{\left(n\right)}-x_{\left(1\right)}},\;x_{\left(n\right)}\;\text{tested}\;\text{if}\;\delta >0\;\;\text{or}\;\;\varepsilon >1 & \\ \frac{x_{\left(2\right)}-x_{\left(1\right)}}{x_{\left(n\right)}-x_{\left(1\right)}},\;x_{\left(1\right)}\;\text{tested}\;\text{if}\;\delta <0\;\text{or}\;\varepsilon <1 & \\ \mathrm{Max}\left(\frac{x_{\left(n\right)}-x_{\left(n-1\right)}}{x_{\left(n\right)}-x_{\left(1\right)}},\frac{x_{\left(2\right)}-x_{\left(1\right)}}{x_{\left(n\right)}-x_{\left(1\right)}}\right), & \\ \;\;\;\;\;x_{\left(n\right)}\;\text{or}\;x_{\left(1\right)}\;\text{tested}\;\text{if}\;\delta =0\;\text{or}\;\varepsilon =1. & \end{array} \end{array}$$

The third test was sample skewness N14 as (note that the absolute value is used for evaluation):

(3) TN14={|n1/2{∑i=1n(xi−x−)3}{∑i=1n(xi−x−)2}3/2|,  x(n)  tested  if  δ>0  or  ε>1|n1/2{∑i=1n(xi−x−)3}{∑i=1n(xi−x−)2}3/2|,  x(1)  tested  if  δ<0  or  ε<1|n1/2{∑i=1n(xi−x−)3}{∑i=1n(xi−x−)2}3/2|,  x(n)  tested  if  n1/2{∑i=1n(xi−x−)3}{∑i=1n(xi−x−)2}3/2>0,δ=0  or  ε=1|n1/2{∑i=1n(xi−x−)3}{∑i=1n(xi−x−)2}3/2|,  x(1)  tested  if  n1/2{∑i=1n(xi−x−)3}{∑i=1n(xi−x−)2}3/2<0,  δ=0  or  ε=−1.

Finally, the fourth test was the sample kurtosis N15 as follows:

(4) $$\begin{array}{c} \text{TN}15=\{\begin{array}{cc} \frac{n\left\{\sum_{i=1}^{n}{\left(x_{i}-\overset{-}{x}\right)}^{4}\right\}}{{\left\{\sum_{i=1}^{n}{\left(x_{i}-\overset{-}{x}\right)}^{2}\right\}}^{2}}, & \\ \;\;x_{\left(n\right)}\;\;\text{tested}\;\;\text{if}\;\;\delta >0\;\;\text{or}\;\;\varepsilon >1 & \\ \frac{n\left\{\sum_{i=1}^{n}{\left(x_{i}-\overset{-}{x}\right)}^{4}\right\}}{{\left\{\sum_{i=1}^{n}{\left(x_{i}-\overset{-}{x}\right)}^{2}\right\}}^{2}}, & \\ \;\;x_{\left(1\right)}\;\;\text{tested}\;\;\text{if}\;\;\delta <0\;\;\text{or}\;\;\varepsilon <1 & \\ \frac{n\left\{\sum_{i=1}^{n}{\left(x_{i}-\overset{-}{x}\right)}^{4}\right\}}{{\left\{\sum_{i=1}^{n}{\left(x_{i}-\overset{-}{x}\right)}^{2}\right\}}^{2}}, & \\ \;\;x_{\left(n\right)}\;\;\text{tested}\;\;\text{if}\;\;\left(x_{\left(n\right)}-\overset{-}{x}\right)>\left(\overset{-}{x}-x_{\left(1\right)}\right), & \\ \delta =0\;\;\text{or}\;\;\varepsilon =1 & \\ \frac{n\left\{\sum_{i=1}^{n}{\left(x_{i}-\overset{-}{x}\right)}^{4}\right\}}{{\left\{\sum_{i=1}^{n}{\left(x_{i}-\overset{-}{x}\right)}^{2}\right\}}^{2}}, & \\ \;\;x_{\left(1\right)}\;\;\text{tested}\;\;\text{if}\;\;\left(x_{\left(n\right)}-\overset{-}{x}\right)<\left(\overset{-}{x}-x_{\left(1\right)}\right), & \\ \;\;\delta >0\;\;\text{or}\;\;\varepsilon =-1. & \end{array} \end{array}$$

All tests were applied at a strict 99% confidence level using the new precise and accurate critical values (CV_99_) simulated using Monte Carlo procedure by Verma et al. [19] for N2, N8, and N15 and Verma and Quiroz-Ruiz [20] for N14, which permitted an objective comparison of their performance.

## 3. Monte Carlo Simulations

Random numbers *U*(0,1) uniformly distributed in the interval (0,1) and normal random variates *N*(0,1) were generated from the method summarized by Verma and Quiroz-Ruiz [21]. However, instead of only 10 series or streams of *N*(0,1) as done by Verma and Quiroz-Ruiz [21], a total of 102 different streams of *N*(0,1) were simulated. Similarly, the replications were much more than those used by Verma and Quiroz-Ruiz [21] for generating precise and accurate critical values.

For a data array of size *n*, (*n* − 1) observations *x*_1_, *x*_2_, *x*_3_,…, *x*_*n*−2_, *x*_*n*−1_ were drawn from one stream of *N*(0,1) and the contaminant observation (*x*_*c*_) was added from a different central tendency shifted stream of *N*(0 + *δ*, 1) where *δ* was varied from +0.1 − +20 and −0.1 − −20 or a dispersion shifted distribution *N*(0,1 × *ε*) where *ε* was varied from +1.1 − +200 and −1.1 − −200. The simulation experiments were also carried out for uncontaminated distributions, in which (*n* − 1) observations were taken from one stream of normal random variates *N*(0,1) and an additional observation was incorporated from a different stream with no contamination; that is, *δ* = 0 and *ε* = ±1.

Now, if we were to arrange the complete array from the lowest to the highest observations, the ordered array could be called *x*_(1)_, *x*_(2)_, *x*_(3)_,…, *x*_(*n*−2)_, *x*_(*n*−1)_, *x*_(*n*)_ after Barnett and Lewis [1]. All four tests under evaluation could then be applied to the resulting data array.

If *δ* > 0, *δ* < 0, *ε* > 1, or *ε* < −1 (the contaminant *x*_*c*_ present), two possibilities would arise for the ordered array *x*_(1)_, *x*_(2)_, *x*_(3)_,…, *x*_(*n*−2)_, *x*_(*n*−1)_, *x*_(*n*)_ as follows (Table 1): (i) the contaminant *x*_*c*_ occupies an inner position in the ordered array; that is, *x*_*c*_ < *x*_(*n*)_ if *δ* > 0 or *ε* > 1 or *x*_*c*_ > *x*_(1)_ if *δ* < 0, or *ε* < −1; this array is called a $\overset{-}{C}$ type event and the contaminant *x*_*c*_ was not used in the test statistic; and (ii) the contaminant *x*_*c*_ occupies the extreme position; that is, *x*_*c*_ = *x*_(*n*)_ if *δ* > 0 or *ε* > 1, or *x*_*c*_ = *x*_(1)_ if *δ* < 0, or *ε* < −1; this array was called a *C* type event and the contaminant *x*_*c*_ was used in the test statistic.

To an event of $\overset{-}{C}$ type when any of these four tests (N2, N8, N14, or N15) was applied, the outcome was called either a spurious type II error probability ($\pi_{\bar{D}\;\bar{C}}$) if the test was not significant or a spurious power ($\pi_{D\bar{C}}$) if it was significant (Table 1). For this decision, the calculated test statistic TN (TN2, TN8, TN14, or TN15) for a sample was compared with the respective CV_99_ [19, 20]. If TN ≤ CV_99_, the outcome of the test was considered as not significant; else when TN > CV_99_, the outcome of the test was considered as significant (Table 1).

Similarly, to an event of *C* type, when a discordancy test was applied, the outcome was either a nonspurious type II error probability ($\pi_{\bar{D}C}$) if the test was not significant or a nonspurious power (*π*_*DC*_) if the test was significant (Table 1).

If *δ* = 0 or *ε* = ±1 (the contaminant *x*_*c*_ absent) and a discordancy test was applied to the ordered array *x*_(1)_, *x*_(2)_, *x*_(3)_,…, *x*_(*n*−2)_, *x*_(*n*−1)_, *x*_(*n*)_ to evaluate the extreme observation *x*_(*n*)_ or *x*_(1)_, the outcome would either be a true negative (the respective probability $\pi_{\bar{D}}$) if the test was not significant, that is, if it failed to detect *x*_(*n*)_ or *x*_(1)_ as discordant, or a type I error (probability *π*_*D*_) if the test was significant; that is, it succeeded in detecting *x*_(*n*)_ or *x*_(1)_ as discordant (Table 1).

## 4. Test Performance Criteria

Hayes and Kinsella [7] documented that a good discordancy test would be characterized by a high nonspurious power probability (high *π*_*DC*_), a low spurious power probability (low $\pi_{D\bar{C}}$), and a low nonspurious type II error probability (low $\pi_{\bar{D}C}$).

Hayes and Kinsella [7] defined the Power of Test (*Ω*) as

(5) $$\begin{array}{c} \Omega =\pi_{D\bar{C}}+\pi_{DC}. \end{array}$$

Similarly, they also defined the Test Performance Criterion *π*_*D*∣*C*_ (which is equivalent to the probability P5 of Barnett and Lewis [1]) or the Conditional Power as

(6) $$\begin{array}{c} \pi_{D\;|\;C}=\text{P}5=\frac{\pi_{DC}}{\left(\pi_{DC}+\pi_{\bar{D}C}\right)}. \end{array}$$

## 5. Optimum Replications

**Figure 1:**
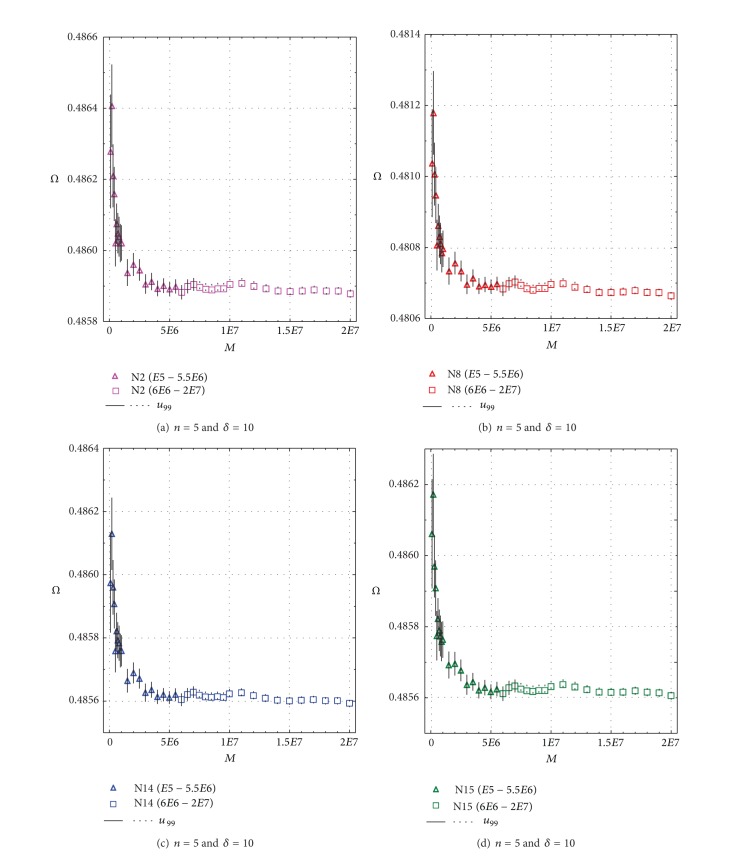
Determination of optimum simulation replication (*M*) for Power of Test (*Ω*) as a function of replications for sample size *n* = 5 and contaminant parameter *δ* = 10; symbols are explained in each figure; the vertical error bars represent uncertainty (*u*_99_) at 99% confidence level from 102 simulations. (a) test N2; (b) test N8; (c) test N14; and (d) test N15.

**Figure 2:**
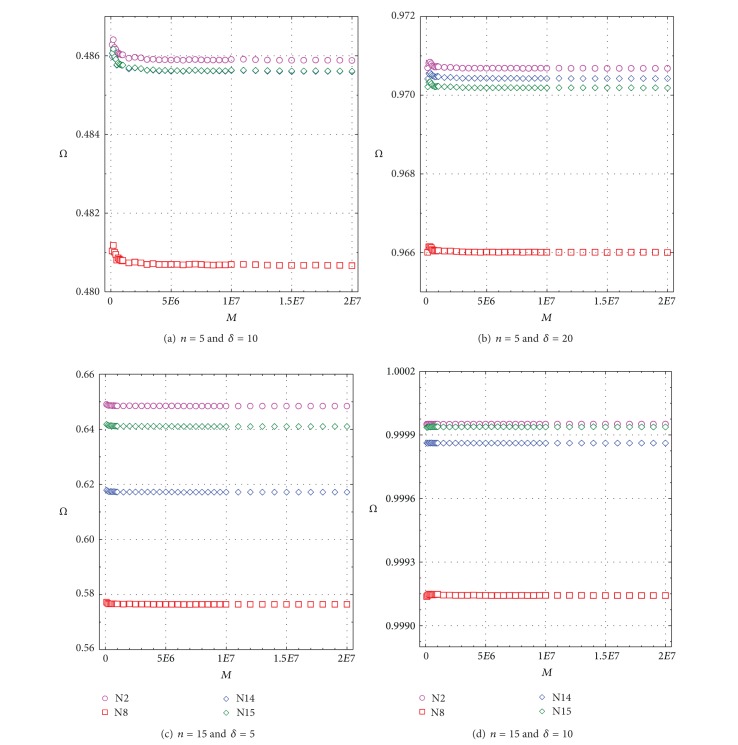
Determination of optimum simulation replication (*M*) for Power of Test (*Ω*) as a function of replications for all tests N2, N8, N14, and N15; symbols are explained in each figure. (a) Sample size *n* = 5 and contaminant parameter *δ* = 10; (b) *n* = 5 and *δ* = 20; (c) *n* = 15 and *δ* = 5; and (d) *n* = 15 and *δ* = 10.

The optimum replications (*M*) required for minimizing the errors of Monte Carlo simulations were decided from representative results summarized in Figures 1 and 2, in which the vertical error bar represents total uncertainty at 99% confidence level (*u*_99_, equivalent to 99% confidence interval of the mean) for 102 simulation experiments. For example, for *n* = 5 and *δ* = 10, Power of Test *Ω* is plotted in Figures 1(a)–1(d) as a function of the replications (*M* = 100,000 to 20,000,000) for N2, N8, N14, and N15. Although *Ω* mean values remain practically constant (within the confidence limits of the mean) for replications of about 8,000,000, still higher replications of 20,000,000 (Figures 1 and 2) were used in all simulation experiments.

Similarly, *Ω* for all four tests as a function of replications is also shown in Figures 2(a)–2(d), which allows a visual comparison of this performance parameter for different sample sizes and *δ* values. Error bars (*u*_99_) for the 102 simulation experiments are not shown for simplicity, but, for replications larger than 10,000,000, they were certainly within the size of the symbols. The replications of 20,000,000 routinely used for comparing the performance of discordancy tests clearly show that the differences among *Ω* values (Figures 2(a)–2(d)) are statistically significant at a high confidence level; that is, these differences are much greater than the simulation errors.

Alternatively, following Krishnamoorthy and Lian [17] the simulation error for the replications of 20,000,000 used routinely in our work can be estimated approximately as $2\times \sqrt{0.5\times 0.5/20000000}=0.00022$.

Because we carried out 102 independent simulation experiments, each with 20,000,000 replications, our simulation errors were even less than the above value. Thus, the Monte Carlo simulations can be considered highly precise. They can also be said to be highly accurate, because our procedure was modified after the highly precise and accurate method of Verma and Quiroz-Ruiz [21]. These authors had shown high precision and accuracy of each *U*(0,1) and *N*(0,1) experiments and had also applied all kinds of simulated data quality tests suggested by Law and Kelton [25]. Besides, in the present work a large number of such experiments (204 streams of *U*(0,1) and 102 streams of *N*(0,1)) have been carried out. Therefore, as an innovation in Monte Carlo simulations we present the mean $\left(\overset{-}{x}\right)$ values as well as the total uncertainty (*u*_99_) of 102 independent experiments in terms of the confidence interval of the mean at the strict 99% confidence level.

Finally, in order to evaluate the test performance, test N2 was used as a reference and differences in mean ($\Delta {\overset{-}{x}}_{\text{N}j}$) values of the other three tests were calculated as

(7) $$\begin{array}{c} \Delta {\overset{-}{x}}_{\text{N}j}=\left(\frac{{\overset{-}{x}}_{\text{N}j}-{\overset{-}{x}}_{\text{N}2}}{{\overset{-}{x}}_{\text{N}2}}\right)\times 100, \end{array}$$

where the subscript N*j* stands for N8, N14, or N15.

## 6. Results and Discussion

### 6.1. $\overset{-}{C}$ Type and Contaminant-Absent Events

**Figure 3:**
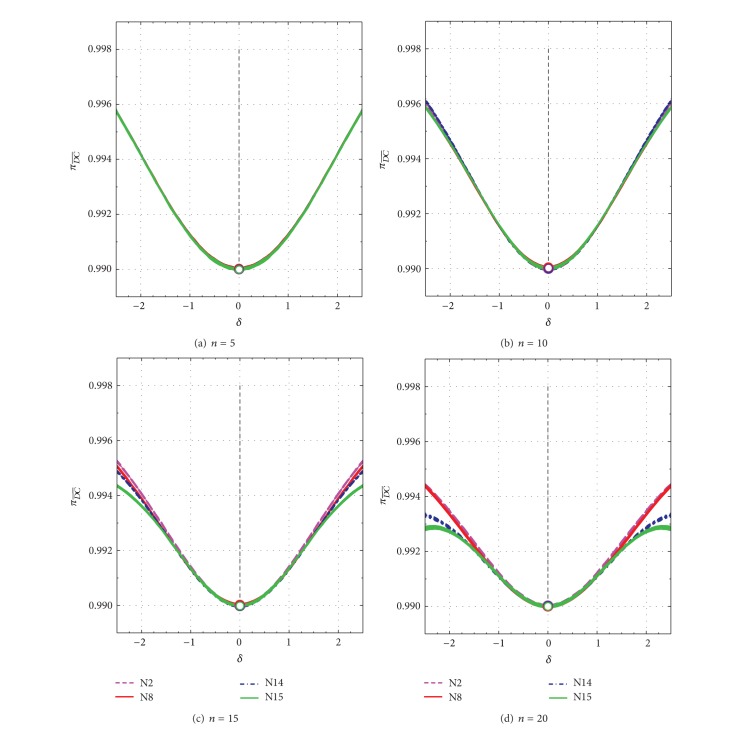
Spurious type II error probability ($\pi_{\bar{D}\;\bar{C}}$) as a function of *δ* from −2.5 to +2.5 for all tests N2, N8, N14, and N15. $\pi_{\bar{D}\;\bar{C}}$ values for uncontaminated samples (*δ* = 0) are shown by open circles. (a) *n* = 5; (b) *n* = 10; (c) *n* = 15; and (d) *n* = 20.

**Figure 4:**
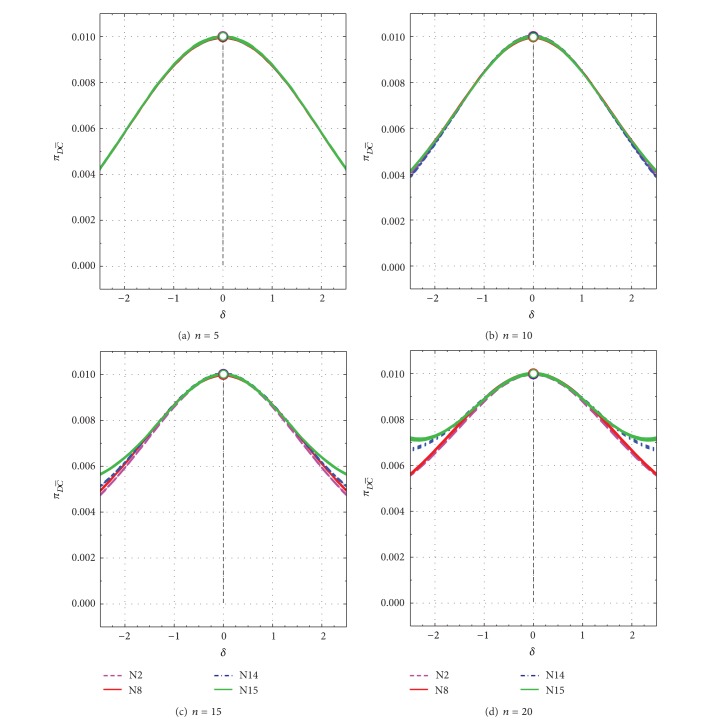
Spurious power probability ($\pi_{D\bar{C}}$) as a function of *δ* from −2.5 to +2.5 for all tests N2, N8, N14, and N15. $\pi_{D\bar{C}}$ values for uncontaminated samples (*δ* = 0) are shown by open circles. (a) *n* = 5; (b) *n* = 10; (c) *n* = 15; and (d) *n* = 20.

According to Barnett and Lewis [1] this type of events is of no major concern, because the contaminant *x*_*c*_ occupies an inner position in the ordered array and the extreme observation *x*_(*n*)_ or *x*_(1)_ under evaluation from discordancy tests is a legitimate observation. An inner position of the contaminant would affect much less the sample mean and standard deviation [1]. For small values of *δ* or *ε* close to 0 or ±1, respectively, most events generated from the Monte Carlo simulation are of $\overset{-}{C}$ type. The $\pi_{\bar{D}\;\bar{C}}$ and $\pi_{D\bar{C}}$ values for *n* = 5 to *n* = 20 as a function of *δ* are presented in Figures 3(a)–3(d) and Figures 4(a)–4(d), respectively. For *ε*, these parameters behave very similarly and, therefore, the corresponding diagrams are not presented.

When the contaminant is absent (*δ* = 0 or *ε* = ±1), the $\pi_{\bar{D}\;\bar{C}}$ and $\pi_{D\bar{C}}$ values are close to the expected values of 0.99 and 0.01, respectively, because the discordancy tests were applied at the 99% confidence level (open circles in Figures 3(a)–3(d) and Figures 4(a)–4(d)). As *δ* changes from 0 to about ±2.5, the $\pi_{\bar{D}\;\bar{C}}$ values slightly increase from 0.99 to about 0.996 for *n* = 5 (Figure 3(a)), 0.996 for *n* = 10 (Figure 3(b)), 0.994-0.995 for *n* = 15 (Figure 3(c)), and 0.993-0.994 for *n* = 20 (Figure 3(d)). The $\pi_{D\bar{C}}$ values show the complementary behavior (Figures 4(a)–4(d)). Because in this type of events $\left(\overset{-}{C}\right)$, a legitimate extreme observation is being tested, our best desire is that the $\pi_{\bar{D}\;\bar{C}}$ and $\pi_{D\bar{C}}$ values remain close to the theoretical values of 0.99 and 0.01, respectively, for contaminant-absent events. This is actually observed in Figures 3 and 4.

### 6.2. *C* Type and Contaminant-Absent Events

**Figure 5:**
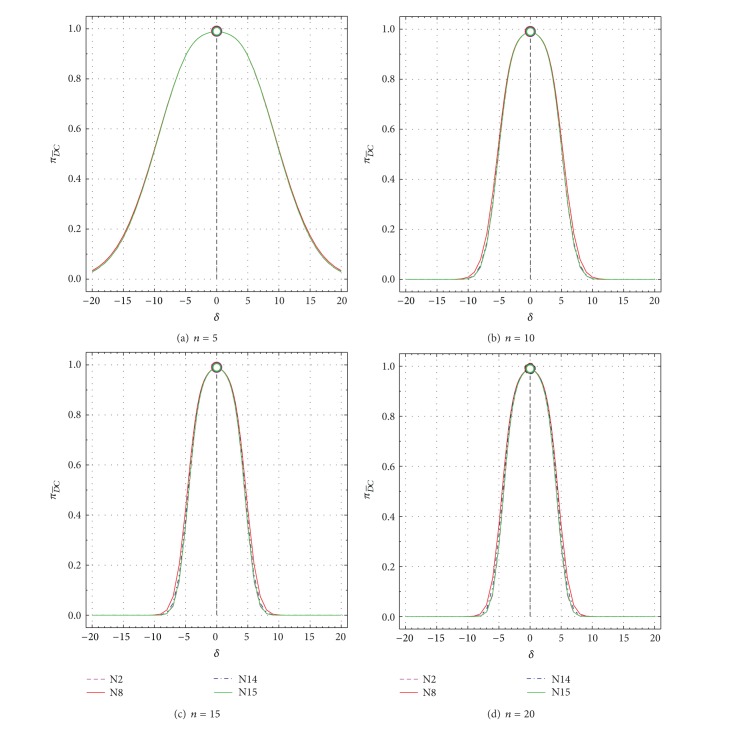
Nonspurious type II error probability ($\pi_{\bar{D}C}$) as a function of *δ* from −20 to +20 for all tests N2, N8, N14, and N15. $\pi_{\bar{D}C}$ values for uncontaminated samples (*δ* = 0) are shown by open circles. (a) *n* = 5; (b) *n* = 10; (c) *n* = 15; and (d) *n* = 20.

**Figure 6:**
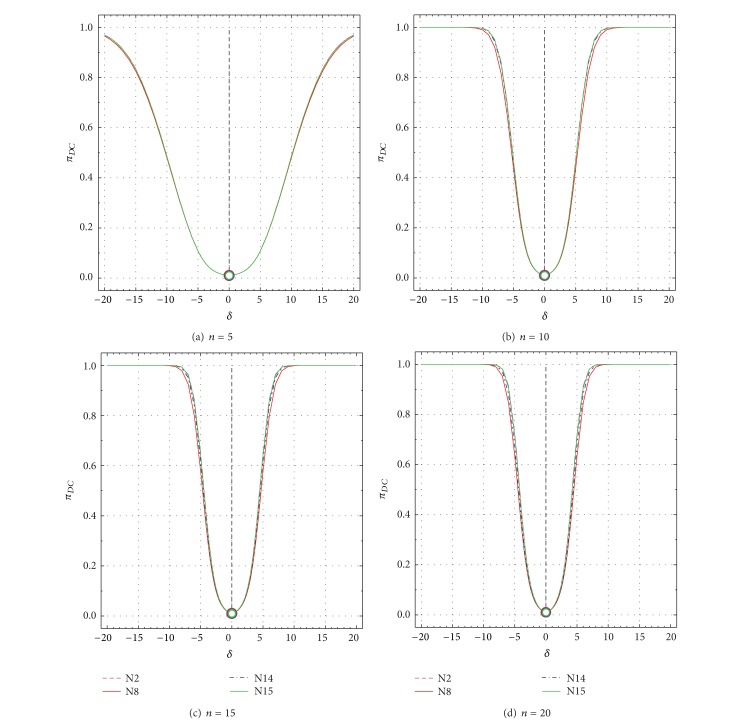
Nonspurious power probability (*π*_*DC*_) as a function of *δ* from −20 to +20 for all tests N2, N8, N14, and N15. *π*_*DC*_ values for uncontaminated samples (*δ* = 0) are shown by open circles. (a) *n* = 5; (b) *n* = 10; (c) *n* = 15; and (d) *n* = 20.

**Figure 7:**
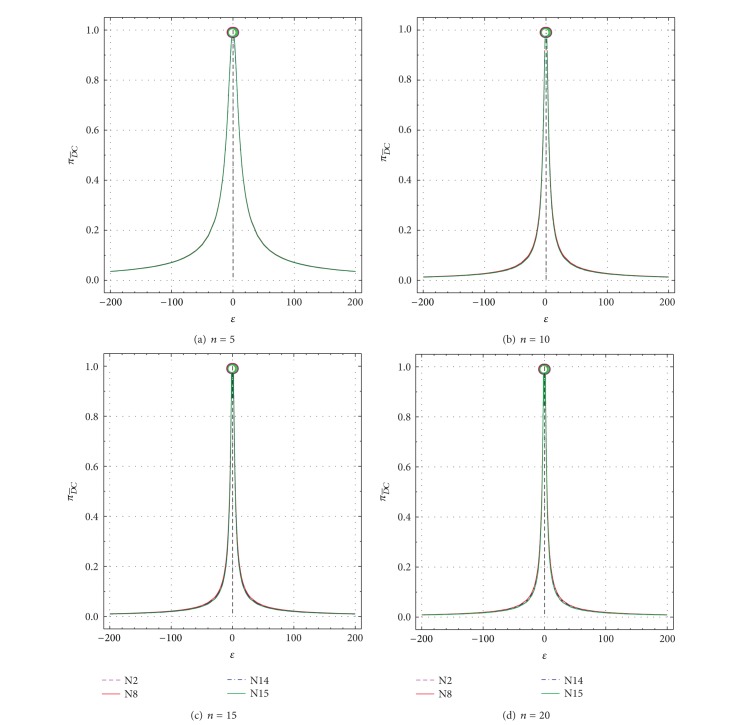
Nonspurious type II error probability ($\pi_{\bar{D}C}$) as a function of *ε* from −1 to −200 and +1 to +200 for all tests N2, N8, N14, and N15. $\pi_{\bar{D}C}$ values for uncontaminated samples (*ε* = ±1) are shown by open circles. (a) *n* = 5; (b) *n* = 10; (c) *n* = 15; and (d) *n* = 20.

**Figure 8:**
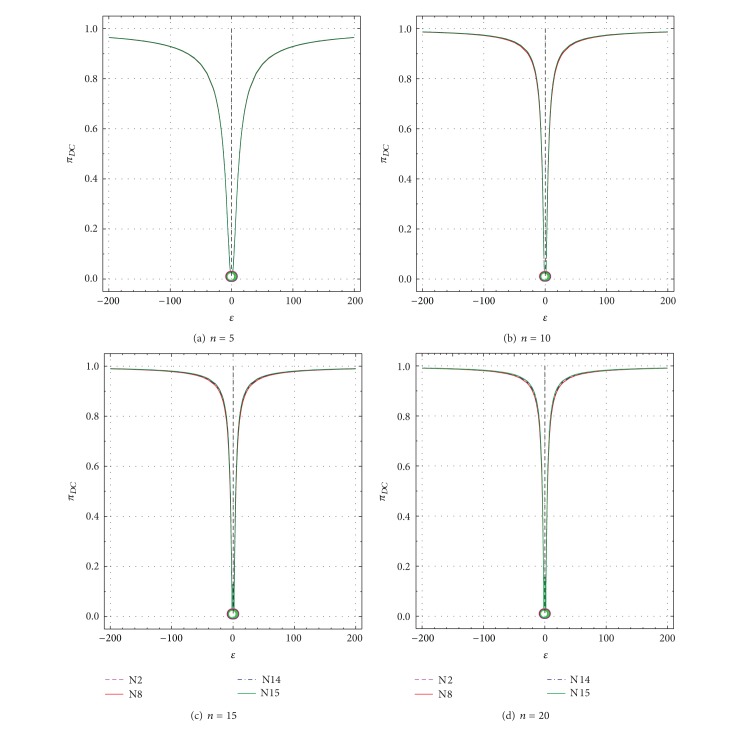
Nonspurious power probability (*π*_*DC*_) as a function of *ε* from −1 to −200 and +1 to +200 for all tests N2, N8, N14, and N15. *π*_*DC*_ values for uncontaminated samples (*ε* = ±1) are shown by open circles. (a) *n* = 5; (b) *n* = 10; (c) *n* = 15; and (d) *n* = 20.

The *C* type events are of major consequence for sample statistical parameters. In such events, because the contaminant *x*_*c*_ occupies an extreme outlying position (*x*_(*n*)_ or *x*_(1)_) in an ordered data array, it is desirable that the discordancy tests detect this contaminant observation as discordant. The $\pi_{\bar{D}C}$ and *π*_*DC*_ values for *n* = 5 to *n* = 20 as a function of *δ* are presented in Figures 5(a)–5(d) and Figures 6(a)–6(d), respectively. Similarly, these values as a function of *ε* are shown in Figures 7(a)–7(d) and Figures 8(a)–8(d), respectively.

For uncontaminated samples (*δ* = 0 in Figures 5(a)–5(d) and Table 2, or *ε* = ±1 in Figures 7(a)–7(d)) the probability $\pi_{\bar{D}C}$ values were close to the theoretical value of 0.99 (which corresponds to the confidence level used for each test). Similarly, for such samples, *π*_*DC*_ values for all sample sizes were close to the theoretical value of 0.01 (complement of 0.99 is 0.01; Figures 6 and 8).

A complementary behavior of $\pi_{\bar{D}C}$ and *π*_*DC*_ exists for all other *δ* or *ε* values as well (Figures 5 and 7 or Figures 6 and 8). Thus, for all tests $\pi_{\bar{D}C}$ decreases sharply from 0.99 for *δ* = 0 to very small values of about 0.03 for *δ* = ±20 and *n* = 5, to about 0.01–0.03 for *δ* = ±9 and *n* = 10, to about 0.006–0.02 for *δ* = ±8 and *n* = 15, and to about 0.001–0.01 for *δ* = ±8 and *n* = 20 (Table 2; Figures 5(a)–5(d)). On the contrary, *π*_*DC*_ increases very rapidly from very small values of 0.01 to close to the maximum theoretical value of 0.99 (see the complementary behavior *π*_*DC*_ in Figures 6(a)–6(d) and Figures 5(a)–5(d)). These probability ($\pi_{\bar{D}C}$ and *π*_*DC*_) values show a similar behavior for larger values of *ε* than for *δ* (compare Figures 7 and 8 with Figures 5 and 6, resp.). There are some differences in these probability values among the different tests (Table 2; Figures 5–8), but they will be better discussed for the test performance criteria.

### 6.3. Test Performance Criteria (*Ω* and *π*_*D*∣*C*_)

**Figure 9:**
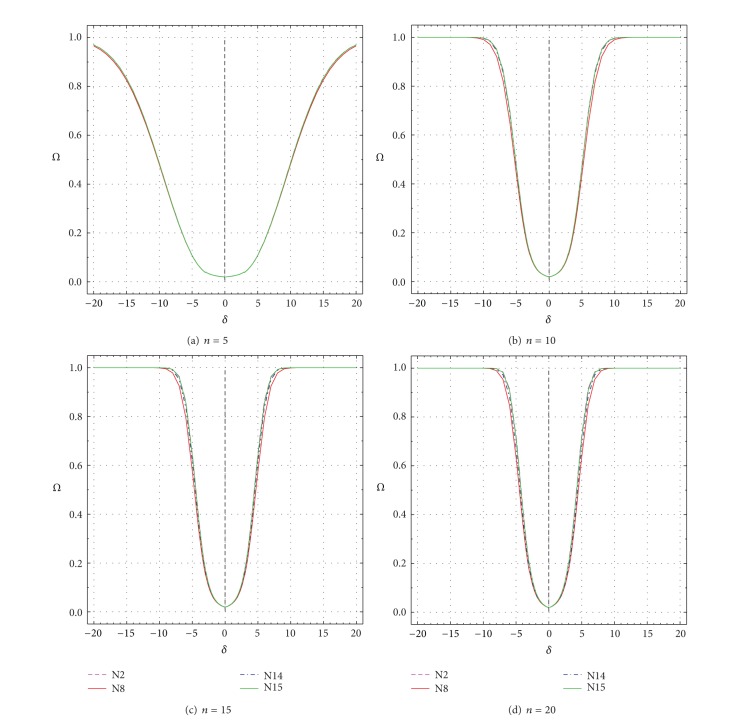
Power of Test (*Ω*) as a function of *δ* from −20 to +20 for all tests N2, N8, N14, and N15. (a) *n* = 5; (b) *n* = 10; (c) *n* = 15; and (d) *n* = 20.

**Figure 10:**
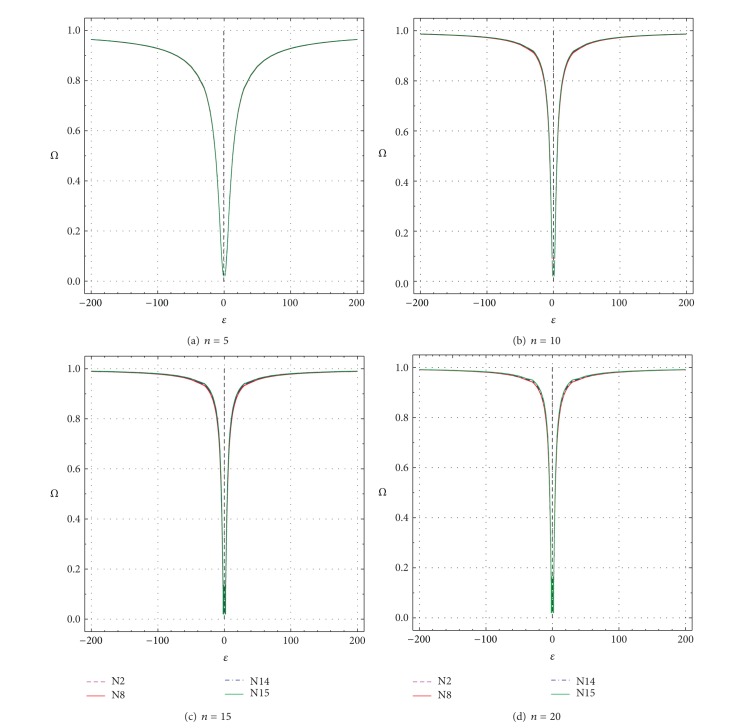
Power of Test (*Ω*) as a function of *ε* from −1 to −200 and +1 to +200 for all tests N2, N8, N14, and N15. (a) *n* = 5; (b) *n* = 10; (c) *n* = 15; and (d) *n* = 20.

**Figure 11:**
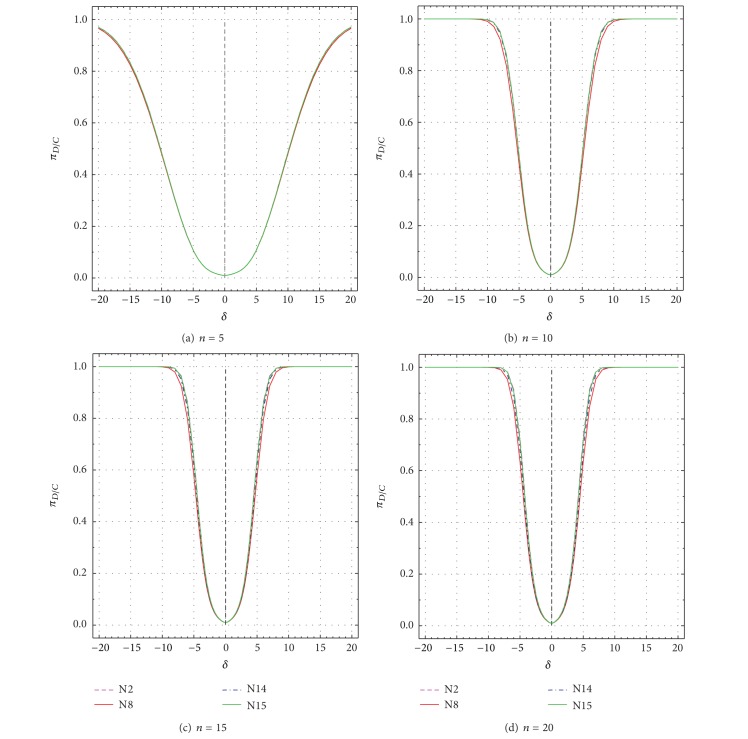
Test Performance Criterion (*π*_*D*∣*C*_, or Conditional Power P5) as a function of *δ* from −20 to +20 for all tests N2, N8, N14, and N15. (a) *n* = 5; (b) *n* = 10; (c) *n* = 15; and (d) *n* = 20.

**Figure 12:**
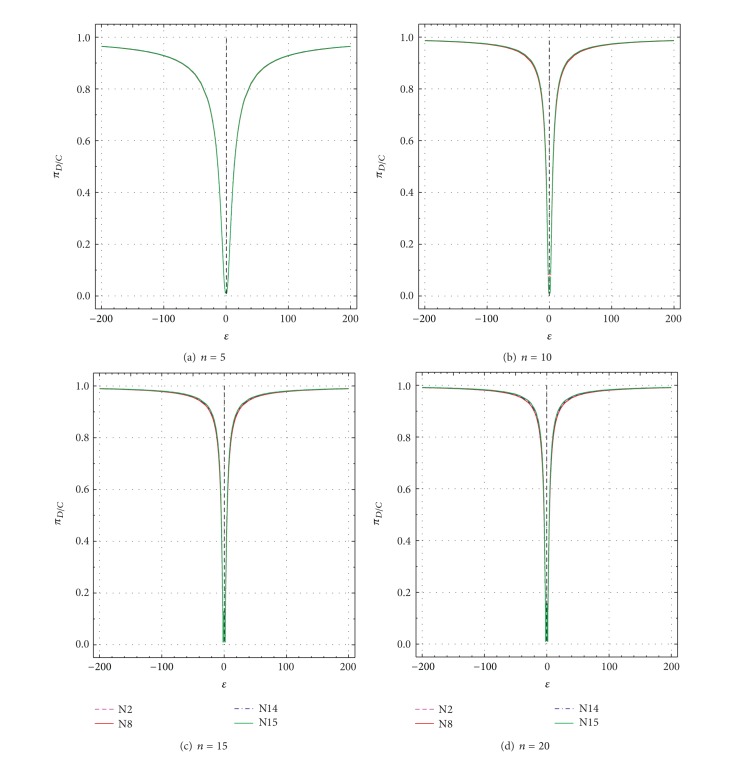
Test Performance Criterion (*π*_*D*∣*C*_, or Conditional Power P5) as a function of *ε* from −1 to −200 and +1 to +200 for all tests N2, N8, N14, and N15. (a) *n* = 5; (b) *n* = 10; (c) *n* = 15; and (d) *n* = 20.

These two parameters are plotted as a function of *δ* and *ε* in Figures 9, 10, 11, and 12 and the most important results are summarized in Tables 3–6. For a good test, both *Ω* ($\pi_{D\bar{C}}+\pi_{DC}$; (5)) and *π*_*D*∣*C*_ (6) should be large [1, 7]. Values of both performance criteria (*Ω* and *π*_*D*∣*C*_) increase as *δ* or *ε* values depart from the uncontaminated values of *δ* = 0 or *ε* = ±1 (Figures 9–12; Tables 3–6). However, *Ω* and *π*_*D*∣*C*_ increase less rapidly for smaller *n* than for larger *n*. For *n* = 5, even for *δ* = ±20 or *ε* = ±200, none of the two parameters truly reaches the maximum theoretical value of 0.99 (Figure 9(a) to Figure 12(a)). For larger *n* (10–20), however, both *Ω* and *π*_*D*∣*C*_ get close to this value for all tests and for much smaller values of *δ* or *ε* than the maximum values of 20 and 200, respectively (Figures 9(b)–9(d) to Figures 12(b)–12(d); Tables 3–6).

The performance differences of the four tests are now briefly discussed in terms of both *δ* and *ε* as well as *n*. The total uncertainty *u*_99_ values of the simulations are extremely small (the error is at the fifth or even sixth decimal place; Tables 3–6). Therefore, most differences among the tests ($\Delta {\overset{-}{x}}_{\text{N}8}$ for test N8, $\Delta {\overset{-}{x}}_{\text{N}14}$ for test N14, and $\Delta {\overset{-}{x}}_{\text{N}15}$ for test N15; all percent differences are with respect to test N2; see (7)) are statistically significant (Tables 3–6). A negative value of $\Delta {\overset{-}{x}}_{\text{N}j}$ (where N*j* stands for N8, N14, or N15) means that *Ω* or *π*_*D*∣*C*_ value for a given test (N8, N14, or N15) is less than that of test N2, implying a worse performance of the given test as compared to test N2, whereas a positive value of $\Delta {\overset{-}{x}}_{\text{N}j}$ signifies just the opposite. Note that test N2 is chosen as a reference test, because it shows generally the best performance (values of $\Delta {\overset{-}{x}}_{\text{N}j}$ are mostly negative in Tables 3–6). Additional fine-scale simulations were also carried out for which both *Ω* and *π*_*D*∣*C*_ become about 0.5 for the reference test N2 (0.5 is about the half of the maximum value of one for *Ω* or *π*_*D*∣*C*_). Hence, the values of *Ω* and *π*_*D*∣*C*_ can be visually compared in Tables 3–6 (see the rows in italic font).

For *n* = 5, all tests show rather similar performance, because the maximum difference ($\Delta {\overset{-}{x}}_{\text{N}j}$) is only about −1.1% for N8 (as compared to N2) and <−0.1% for N14 and N15 (see the first set of rows corresponding to *n* = 5 in Tables 3–6). Test N2 shows *Ω* = 0.50044 for *δ* = ±10.17, whereas tests N8, N14, and N15 have *Ω* values of 0.49503, 0.50014, and 0.50015, respectively, (Table 3). The respective $\Delta {\overset{-}{x}}_{\text{N}j}$ values are about −1.1%, −0.06%, and −0.06% (Table 3). Practically the same results are valid for *π*_*D*∣*C*_ as well (see the row in italic font in Table 4). Similar results were documented for *Ω* and *π*_*D*∣*C*_ as a function of *ε* (rows for *ε* = ±12.9 or ±13.1 in Tables 5 and 6, resp.).

For *n* = 10, Dixon test N8 becomes considerably less efficient than Grubbs test N2, because the $\Delta {\overset{-}{x}}_{\text{N}8}$ values become as low as −7.8% for *δ* = ±5 or −6.4% for *ε* = ±3 (Tables 3–6). Skewness test N14 also shows slightly lower *Ω* and *π*_*D*∣*C*_ than N2 ($\Delta {\overset{-}{x}}_{\text{N}14}=-1.8$% for *δ* = ±4- ± 5, or $\Delta {\overset{-}{x}}_{\text{N}14}=-1.5$% for *ε* = ±3; Tables 3–6). Kurtosis test N15 shows a similar performance as test N2; the maximum difference $\Delta {\overset{-}{x}}_{\text{N}15}$ is about 0.7 (Tables 3–6). For *n* = 10 test N2 shows *Ω* = 0.5 (or *π*_*D*∣*C*_ = 0.5) for *δ* = ±5.105; for this case, the other three tests (N8, N14, and N15) show $\Delta {\overset{-}{x}}_{\text{N}j}$ values of about −7.8%, −1.8%, and −0.7% (Tables 3 and 4). Similarly, for such cases, *Ω* and *π*_*D*∣*C*_ show $\Delta {\overset{-}{x}}_{\text{N}8}$, $\Delta {\overset{-}{x}}_{\text{N}14}$, and $\Delta {\overset{-}{x}}_{\text{N}15}$ values of about −4.3%, −1.0%, and −0.4%, respectively.

For *n* = 15 and *n* = 20, test N8 shows the worst performance and the $\Delta {\overset{-}{x}}_{\text{N}8}$ values become as large as −12.2% to −15.5% for *δ* (Tables 3 and 4) or −9.8% to −11.5% for *ε* (Tables 5 and 6). For these sample sizes, test N14 also shows a worse performance as compared to N2, because the maximum differences represented by $\Delta {\overset{-}{x}}_{\text{N}14}$ values are about −6.3% to −10.9% for *δ* (Tables 3 and 4) or −4.5% to −7.0% for *ε* (Tables 5 and 6). Test N15 shows a comparable performance, because the maximum differences ($\Delta {\overset{-}{x}}_{\text{N}15}$ values) are about −1.5% to −2.4% for *δ* (Tables 3 and 4) or −1.1% to −1.5% for *ε* (Tables 5 and 6). For *n* = 15 and *n* = 20, when test N2 shows *Ω* = 0.5 or *π*_*D*∣*C*_ = 0.5, the $\Delta {\overset{-}{x}}_{\text{N}8}$, $\Delta {\overset{-}{x}}_{\text{N}14}$, and $\Delta {\overset{-}{x}}_{\text{N}15}$ values range from about −6.9% to −15.0%, −3.0% to −9.2%, and −0.6% to −1.9%, respectively.

The significantly lower *Ω* and *π*_*D*∣*C*_ values of the Dixon test N8 as compared to the Grubbs test N2, skewness test N14, and kurtosis test N15 may be related to the masking effect of the penultimate observation *x*_(*n*−1)_ on *x*_(*n*)_ or of *x*_(2)_ on *x*_(1)_ as documented by Barnett and Lewis [1]. The masking effect may also be responsible for a somewhat worse performance of N14 as compared to N2.

### 6.4. Final Remarks

The two performance criteria (*Ω* and *π*_*D*∣*C*_) [1, 7] used in this work provide similar estimates (Tables 3–6) and, more importantly, similar conclusions. Therefore, any of them can be used to evaluate numerous other discordancy tests for single or multiple outliers [1, 26–28]. The main result of Monte Carlo simulations concerning the performance of the single extreme outlier discordancy tests could be stated as follows: N2≅N15 > N14 > N8.

Additional simulation work is required to evaluate other discordancy tests, such as the single upper or lower outlier tests, as well as more complex statistical contamination involving two or more discordant outliers and the comparison of consecutive application of single outlier discordancy tests with multiple outlier tests [1, 7, 26–28]. Then, the multiple test method, initially proposed by Verma [29] and used by many researchers [30–35], would be substantially improved for subsequent applications. These performance results could then be incorporated in new versions of the computer programs DODESSYS [36], TecD [37], and UDASYS [38].

## 7. Conclusions

Our simulation study clearly shows that Dixon test N8 performs less well than the other three extreme single outlier tests (Grubbs N2, skewness N14, and kurtosis N15). Both performance parameters (the Power of Test *Ω* and Test Performance Criterion *π*_*D*∣*C*_) have up to about 16% less values for N8 than test N2. Test N8, therefore, shows the worst performance for outlier detection. For certain values of *δ* or *ε* test N14 also shows lesser values of *Ω* and *π*_*D*∣*C*_ than N2, which means that N14 is also somewhat worse than N2. The other two tests (N2 and N15) could be considered comparable in their performance.

## Figures and Tables

**Table 1:** Sample simulation and test outcome (modified after Hayes and Kinsella [7]).

Discordancy test result	Contaminant *x*_*c*_ (*M* replications)		
	Present (*δ*> 0, *δ*< 0, *ε* > 1, or *ε*< −1)		Absent (*δ* = 0 or *ε* = 1)
	Contaminant *x*_*c*_ not used in a discordancy test ($\overset{-}{C}$ type event)	Contaminant *x*_*c*_ used in a discordancy test (*C* type event)	
Not significant (TN ≤ CV_99_)	Spurious type II error probability ($\pi_{\bar{D}\bar{C}}$) (Figure 3)	Nonspurious type II error probability ($\pi_{\bar{D}C}$) (Figures 5 and 7)	True negative probability ($\pi_{\bar{D}}$) (Figures 3, 5, and 7)

Significant (TN > CV_99_)	Spurious power probability ($\pi_{D\bar{C}}$) (Figure 4)	Nonspurious power probability (π_*DC*_) (Figures 6 and 8)	Type I error probability (π_*D*_) (Figures 4, 6, and 8)

TN: calculated test statistic for a sample (TN2, TN8, TN14, or TN15); CV_99_: critical value for a given discordancy test at 99% confidence level.

**Table 2:** NonSpurious type II error probability ($\pi_{\bar{D}C}$) parameter for four single extreme outlier discordancy tests.

*n*	*δ*	Discordancy tests										
		N2		N8			N14			N15		
		${\overset{-}{x}}_{\text{N2}}$	(*u*_99_)_N2_	${\overset{-}{x}}_{\text{N8}}$	(*u*_99_)_N8_	$\Delta {\overset{-}{x}}_{\text{N8}}$	${\overset{-}{x}}_{\text{N14}}$	(*u*_99_)_N14_	$\Delta {\overset{-}{x}}_{\text{N14}}$	${\overset{-}{x}}_{\text{N15}}$	(*u*_99_)_N15_	$\Delta {\overset{-}{x}}_{\text{N15}}$
5	0	0.990003	0.0000057	0.990026	0.0000058	0.0023	0.990005	0.0000057	0.0002	0.989994	0.0000058	−0.0009
5	±0.1	0.989948	0.0000139	0.989975	0.0000140	0.0027	0.989950	0.0000140	0.0002	0.989940	0.0000144	−0.0009
5	±2	0.977285	0.0000113	0.977358	0.0000125	0.0075	0.977290	0.0000111	0.0005	0.977267	0.0000116	−0.0018
5	±3	0.961486	0.0000148	0.961632	0.0000142	0.0152	0.961497	0.0000146	0.0012	0.961459	0.0000146	−0.0028
5	±5	0.891864	0.0000205	0.892491	0.0000194	0.070	0.891901	0.0000200	0.0043	0.891823	0.0000208	−0.0046
5	±6	0.834635	0.0000222	0.835779	0.0000231	0.137	0.834704	0.0000226	0.0084	0.834602	0.0000225	−0.0038
5	±7	0.764660	0.0000240	0.766559	0.0000257	0.248	0.764766	0.0000250	0.0139	0.764661	0.0000237	0.0002
5	±8	0.685336	0.0000253	0.688202	0.0000255	0.418	0.685496	0.0000249	0.0234	0.685401	0.0000247	0.0095
5	±10	0.514122	0.0000280	0.519336	0.0000282	1.01	0.514407	0.0000292	0.056	0.514394	0.0000287	0.053
5	±12	0.351103	0.0000258	0.358468	0.0000275	2.10	0.351500	0.0000257	0.113	0.351629	0.0000258	0.150
5	±15	0.165615	0.0000194	0.174053	0.0000215	5.1	0.166070	0.0000196	0.275	0.166367	0.0000196	0.45
5	±18	0.062959	0.0000126	0.069566	0.0000148	10.5	0.063321	0.0000127	0.58	0.063618	0.0000134	1.05
5	±20	0.029322	0.0000082	0.033997	0.0000089	15.9	0.029577	0.0000084	0.87	0.029815	0.0000081	1.68
10	0	0.990008	0.0000057	0.990041	0.0000057	0.0034	0.990003	0.0000054	−0.0006	0.990025	0.0000059	0.0017
10	±0.1	0.989851	0.0000197	0.989888	0.0000173	0.0037	0.989844	0.0000207	−0.0006	0.989869	0.0000195	0.0019
10	±1	0.978810	0.0000183	0.979321	0.0000174	0.052	0.978911	0.0000179	0.0103	0.978879	0.0000188	0.0070
10	±2	0.949169	0.0000168	0.951501	0.0000163	0.246	0.949704	0.0000165	0.056	0.949433	0.0000175	0.0279
10	±3	0.878974	0.0000199	0.886717	0.0000192	0.88	0.880804	0.0000197	0.208	0.879762	0.0000202	0.090
10	±4	0.736953	0.0000241	0.756911	0.0000242	2.71	0.741679	0.0000266	0.64	0.738849	0.0000253	0.257
10	±5	0.523889	0.0000273	0.561084	0.0000257	7.1	0.532523	0.0000263	1.65	0.527197	0.0000266	0.63
10	±6	0.300940	0.0000248	0.349468	0.0000258	16.1	0.311799	0.0000260	3.61	0.304921	0.0000249	1.32
10	±7	0.136560	0.0000182	0.181585	0.0000226	33.0	0.146058	0.0000174	7.0	0.139899	0.0000185	2.44
10	±8	0.048488	0.0000122	0.079155	0.0000144	63	0.054422	0.0000118	12.2	0.050495	0.0000130	4.14
10	±9	0.013431	0.0000074	0.029234	0.0000092	118	0.016139	0.0000075	20.2	0.014300	0.0000074	6.5
10	±10	0.002900	0.0000031	0.009248	0.0000056	219	0.003821	0.0000036	31.8	0.003180	0.0000030	9.6
10	±11	0.000489	0.0000013	0.002529	0.0000029	418	0.000726	0.0000016	49	0.000556	0.0000014	13.8
15	0	0.989998	0.000006	0.990015	0.0000062	0.0017	0.98998	0.0000061	−0.0018	0.989989	0.000006	−0.0010
15	±0.1	0.989768	0.0000204	0.989811	0.0000210	0.0044	0.989766	0.0000209	−0.0002	0.989759	0.0000200	−0.0009
15	±0.5	0.985307	0.0000236	0.985665	0.0000202	0.0364	0.985507	0.0000249	0.0204	0.985345	0.0000240	0.0039
15	±2	0.974943	0.0000225	0.976229	0.0000186	0.132	0.975702	0.0000241	0.078	0.975113	0.0000236	0.0174
15	±5	0.932551	0.0000202	0.938851	0.0000184	0.68	0.936096	0.0000207	0.380	0.933417	0.0000206	0.093
15	±4	0.827672	0.0000245	0.848433	0.0000236	2.51	0.838586	0.0000251	1.32	0.830350	0.0000236	0.324
15	±4.5	0.621647	0.0000288	0.669717	0.0000270	7.7	0.644852	0.0000332	3.73	0.627283	0.0000285	0.91
15	±5	0.487591	0.0000276	0.550018	0.0000319	12.8	0.516243	0.0000322	5.9	0.494476	0.0000290	1.41
15	±5	0.351577	0.0000269	0.423601	0.0000266	20.5	0.382834	0.0000290	8.9	0.358972	0.0000287	2.10
15	±6	0.137443	0.0000198	0.204194	0.0000211	49	0.162735	0.0000196	18.4	0.143150	0.0000201	4.15
15	±7	0.035757	0.0000093	0.074779	0.0000144	109	0.048090	0.0000112	34.5	0.038352	0.0000098	7.3
15	±8	0.006107	0.0000043	0.021184	0.0000094	249	0.009822	0.0000054	61	0.006819	0.0000042	11.7
15	±9	0.000682	0.0000015	0.004748	0.0000039	600	0.001393	0.0000019	104	0.000802	0.0000017	17.6
15	±10	0.000049	0.0000004	0.000858	0.0000017	1640	0.000138	0.0000007	180	0.000062	0.0000005	25.7
20	0	0.990017	0.0000057	0.990004	0.000006	−0.0014	0.990026	0.000006	0.0009	0.990004	0.0000059	−0.0014
20	±0.1	0.989743	0.0000259	0.989771	0.0000248	0.0028	0.989786	0.0000243	0.0043	0.989734	0.0000277	−0.0009
20	±0.5	0.984600	0.0000243	0.985127	0.0000258	0.054	0.985093	0.0000265	0.050	0.984682	0.0000246	0.0083
20	±1	0.972754	0.0000237	0.974729	0.0000256	0.203	0.974439	0.0000213	0.173	0.973084	0.0000225	0.0339
20	±2	0.922859	0.0000259	0.932611	0.0000219	1.06	0.930457	0.0000234	0.82	0.924463	0.0000266	0.174
20	±3	0.798422	0.0000265	0.829677	0.0000230	3.91	0.820494	0.0000248	2.76	0.803167	0.0000283	0.59
20	±4	0.561409	0.0000283	0.628839	0.0000267	12.0	0.604043	0.0000273	7.6	0.570474	0.0000288	1.61
20	±4.5	0.415514	0.0000280	0.498225	0.0000261	19.9	0.464616	0.0000262	11.8	0.425784	0.0000285	2.47
20	±5	0.276626	0.0000244	0.365289	0.0000265	32.1	0.325675	0.0000297	17.7	0.286659	0.0000245	3.63
20	±6	0.085535	0.0000163	0.152881	0.0000198	79	0.116833	0.0000185	36.6	0.091506	0.0000175	7.0
20	±7	0.015809	0.0000070	0.046048	0.0000127	191	0.026842	0.0000087	70	0.017699	0.0000079	12.0
20	±8	0.001716	0.0000021	0.010242	0.0000053	500	0.003921	0.0000036	128	0.002041	0.0000025	18.9
20	±9	0.000109	0.0000006	0.001733	0.0000024	1500	0.000366	0.0000011	237	0.000139	0.0000007	28.5
20	±10	0.000004	0.0000001	0.000229	0.0000010	5600	0.000022	0.0000002	450	0.000006	0.0000001	41.3

**Table 3:** Power of Test (Ω) values for four single extreme outlier discordancy tests as a function of *δ*.

*n*	*δ*	Discordancy tests										
		N2		N8			N14			N15		
		${\overset{-}{x}}_{\text{N2}}$	(*u*_99_)_N2_	${\overset{-}{x}}_{\text{N8}}$	(*u*_99_)_N8_	$\Delta {\overset{-}{x}}_{\text{N8}}$	${\overset{-}{x}}_{\text{N14}}$	(*u*_99_)_N14_	$\Delta {\overset{-}{x}}_{\text{N14}}$	${\overset{-}{x}}_{\text{N15}}$	(*u*_99_)_N15_	$\Delta {\overset{-}{x}}_{\text{N15}}$
5	±0.1	0.020034	0.0000163	0.019986	0.0000163	−0.243	0.020031	0.0000165	−0.0191	0.020053	0.0000167	0.092
5	±2	0.028537	0.0000125	0.028456	0.0000128	−0.283	0.028531	0.0000124	−0.0224	0.028563	0.0000127	0.089
5	±3	0.038514	0.0000148	0.038368	0.0000142	−0.380	0.038503	0.0000146	−0.0291	0.038541	0.0000146	0.070
5	±4	0.065920	0.0000155	0.065606	0.0000154	−0.48	0.065900	0.0000155	−0.0309	0.065955	0.0000157	0.054
5	±5	0.108136	0.0000205	0.107509	0.0000194	−0.58	0.108099	0.0000200	−0.0351	0.108177	0.0000208	0.0379
5	±6	0.165365	0.0000222	0.164221	0.0000231	−0.69	0.165296	0.0000226	−0.0422	0.165398	0.0000225	0.0194
5	±7	0.235340	0.0000240	0.233441	0.0000257	−0.81	0.235234	0.0000250	−0.045	0.235339	0.0000237	−0.0007
5	±8	0.314664	0.0000253	0.311798	0.0000255	−0.91	0.314504	0.0000249	−0.051	0.314599	0.0000247	−0.0206
5	±10	0.485878	0.0000280	0.480664	0.0000282	−1.07	0.485593	0.0000292	−0.059	0.485606	0.0000287	−0.056
***5***	***±10.17***	***0.50044***	***0.0000279***	***0.49503***	***0.0000287***	***−1.08***	***0.50014***	***0.0000280***	***−0.059***	***0.50015***	***0.0000280***	***−0.058***
5	±12	0.648897	0.0000258	0.641532	0.0000275	−1.14	0.648500	0.0000257	−0.061	0.648371	0.0000258	−0.081
5	±15	0.834385	0.0000194	0.825947	0.0000215	−1.01	0.833930	0.0000196	−0.055	0.833633	0.0000196	−0.090
5	±18	0.937041	0.0000126	0.930434	0.0000148	−0.71	0.936679	0.0000127	−0.0387	0.936382	0.0000134	−0.070
5	±20	0.970678	0.0000082	0.966003	0.0000089	−0.48	0.970423	0.0000084	−0.0263	0.970185	0.0000081	−0.051
10	±0.1	0.020121	0.0000216	0.020050	0.0000181	−0.352	0.020131	0.0000218	0.052	0.020087	0.0000211	−0.169
10	±1	0.029615	0.0000189	0.029124	0.0000180	−1.66	0.029522	0.0000185	−0.316	0.029546	0.0000193	−0.233
10	±2	0.056182	0.0000169	0.053967	0.0000177	−3.94	0.055608	0.0000161	−1.02	0.056005	0.0000176	−0.314
10	±3	0.121026	0.0000199	0.113283	0.0000192	−6.4	0.119196	0.0000197	−1.51	0.120238	0.0000202	−0.65
10	±4	0.263047	0.0000241	0.243089	0.0000242	−7.6	0.258321	0.0000266	−1.80	0.261151	0.0000253	−0.72
10	±5	0.476111	0.0000273	0.438916	0.0000257	−7.8	0.467477	0.0000263	−1.81	0.472803	0.0000266	−0.69
***10***	***±5.105***	***0.500474***	***0.0000285***	***0.461588***	***0.0000261***	−***7.8***	***0.491473***	***0.0000273***	−***1.80***	***0.497048***	***0.0000272***	−***0.68***
10	±6	0.699060	0.0000248	0.650532	0.0000258	−6.9	0.688201	0.0000260	−1.55	0.695079	0.0000249	−0.57
10	±7	0.863440	0.0000182	0.818415	0.0000226	−5.2	0.853942	0.0000174	−1.10	0.860101	0.0000185	−0.387
10	±8	0.951512	0.0000122	0.920845	0.0000144	−3.22	0.945578	0.0000118	−0.62	0.949505	0.0000130	−0.211
10	±9	0.986569	0.0000074	0.970766	0.0000092	−1.60	0.983861	0.0000075	−0.274	0.985700	0.0000074	−0.088
10	±10	0.997100	0.0000031	0.990752	0.0000056	−0.64	0.996179	0.0000036	−0.092	0.996820	0.0000030	−0.0280
10	±11	0.999511	0.0000013	0.997471	0.0000029	−0.204	0.999274	0.0000016	−0.0237	0.999444	0.0000014	−0.0067
15	±0.1	0.020212	0.0000214	0.020157	0.0000214	−0.272	0.020236	0.0000217	0.117	0.020233	0.0000212	0.106
15	±0.5	0.024306	0.0000241	0.023963	0.0000203	−1.41	0.024143	0.0000259	−0.67	0.024284	0.0000250	−0.091
15	±1	0.033660	0.0000236	0.032450	0.0000192	−3.60	0.032982	0.0000251	−2.01	0.033545	0.0000245	−0.341
15	2.5	0.113807	0.0000218	0.102067	0.0000195	−10.3	0.107680	0.0000210	−5.4	0.113112	0.0000226	−0.61
15	±3	0.172328	0.0000245	0.151567	0.0000236	−12.0	0.161414	0.0000251	−6.3	0.169650	0.0000236	−1.55
15	±4	0.378353	0.0000288	0.330283	0.0000270	−12.7	0.355148	0.0000332	−6.1	0.372717	0.0000285	−1.49
***15***	***±4.46***	***0.501373***	***0.0000279***	***0.439974***	***0.0000313***	−***12.2***	***0.473072***	***0.0000321***	−***5.6***	***0.494564***	***0.0000297***	−***1.36***
15	±5	0.648423	0.0000269	0.576399	0.0000266	−11.1	0.617166	0.0000290	−4.8	0.641028	0.0000287	−1.14
15	±6	0.862557	0.0000198	0.795806	0.0000211	−7.74	0.837265	0.0000196	−2.93	0.856850	0.0000201	−0.66
15	±7	0.964243	0.0000093	0.925221	0.0000144	−4.05	0.951910	0.0000112	−1.28	0.961648	0.0000098	−0.269
15	±8	0.993893	0.0000043	0.978816	0.0000094	−1.52	0.990178	0.0000054	−0.374	0.993181	0.0000042	−0.072
15	±9	0.999318	0.0000015	0.995252	0.0000039	−0.407	0.998607	0.0000019	−0.071	0.999198	0.0000017	−0.0120
15	±10	0.999951	0.0000004	0.999142	0.0000017	−0.081	0.999862	0.0000007	−0.0089	0.999938	0.0000005	−0.00127
20	±0.1	0.020222	0.0000253	0.020213	0.0000242	−0.046	0.020175	0.0000238	−0.237	0.020248	0.0000270	0.126
20	±0.5	0.025049	0.0000251	0.024566	0.0000260	−1.93	0.024578	0.0000267	−1.88	0.024992	0.0000255	−0.227
20	±1	0.036024	0.0000249	0.034154	0.0000266	−5.2	0.034454	0.0000228	−4.36	0.035781	0.0000235	−0.67
20	2.5	0.132180	0.0000257	0.113955	0.0000238	−13.8	0.119739	0.0000238	−9.4	0.130861	0.0000269	−1.00
20	±3	0.201578	0.0000265	0.170323	0.0000230	−15.5	0.179506	0.0000248	−10.9	0.196833	0.0000283	−2.35
20	4	0.438591	0.0000283	0.374722	0.0000286	−14.6	0.395957	0.0000273	−9.7	0.429526	0.0000288	−2.07
***20***	***±4.21***	***0.499301***	***0.0000272***	***0.424653***	***0.0000266***	−***15.0***	***0.453296***	***0.0000268***	−***9.2***	***0.489576***	***0.0000279***	−***1.95***
20	±5	0.723374	0.0000244	0.634711	0.0000265	−12.3	0.674325	0.0000297	−6.8	0.713341	0.0000245	−1.39
20	±6	0.914465	0.0000163	0.847119	0.0000198	−7.4	0.883167	0.0000185	−3.42	0.908494	0.0000175	−0.65
20	±7	0.984191	0.0000070	0.953952	0.0000127	−3.07	0.973158	0.0000087	−1.12	0.982301	0.0000079	−0.192
20	±8	0.998284	0.0000021	0.989758	0.0000053	−0.85	0.996079	0.0000036	−0.221	0.997959	0.0000025	−0.0325
20	±9	0.999891	0.0000006	0.998267	0.0000024	−0.162	0.999634	0.0000011	−0.0258	0.999861	0.0000007	−0.00309
20	±10	0.999996	0.0000001	0.999771	0.0000010	−0.0225	0.999978	0.0000002	−0.00180	0.999994	0.0000001	−0.00017

**Table 4:** Test Performance Criterion P5 (π_*D*∣*C*_) values for four single extreme outlier discordancy tests as a function of *δ*.

*n*	*δ*	Discordancy tests										
		N2		N8			N14			N15		
		${\overset{-}{x}}_{\text{N2}}$	(*u*_99_)_N2_	${\overset{-}{x}}_{\text{N8}}$	(*u*_99_)_N8_	$\Delta {\overset{-}{x}}_{\text{N8}}$	${\overset{-}{x}}_{\text{N14}}$	(*u*_99_)_N14_	$\Delta {\overset{-}{x}}_{\text{N14}}$	${\overset{-}{x}}_{\text{N15}}$	(*u*_99_)_N15_	$\Delta {\overset{-}{x}}_{\text{N15}}$
5	±0.1	0.010052	0.0000139	0.010025	0.0000140	−0.262	0.010050	0.0000140	−0.0153	0.010060	0.0000144	0.085
5	±2	0.022715	0.0000113	0.022642	0.0000125	−0.322	0.022710	0.0000111	−0.0212	0.022733	0.0000116	0.079
5	±3	0.038514	0.0000148	0.038368	0.0000142	−0.380	0.038503	0.0000146	−0.0291	0.038541	0.0000146	0.070
5	±4	0.065920	0.0000155	0.065606	0.0000154	−0.48	0.065900	0.0000155	−0.0309	0.065955	0.0000157	0.054
5	±5	0.108136	0.0000205	0.107509	0.0000194	−0.58	0.108099	0.0000200	−0.0351	0.108177	0.0000208	0.0379
5	±6	0.165365	0.0000222	0.164221	0.0000231	−0.69	0.165296	0.0000226	−0.0422	0.165398	0.0000225	0.0194
5	±7	0.235340	0.0000240	0.233441	0.0000257	−0.81	0.235234	0.0000250	−0.045	0.235339	0.0000237	−0.00066
5	±8	0.314664	0.0000253	0.311798	0.0000255	−0.91	0.314504	0.0000249	−0.051	0.314599	0.0000247	−0.0206
5	±10	0.485878	0.0000280	0.480664	0.0000282	−1.07	0.485593	0.0000292	−0.059	0.485606	0.0000287	−0.056
***5***	***±10.17***	***0.50044***	***0.0000279***	***0.49503***	***0.0000287***	−***1.08***	***0.50014***	***0.0000280***	−***0.059***	***0.50015***	***0.0000280***	−***0.058***
5	±12	0.648897	0.0000258	0.641532	0.0000275	−1.14	0.648500	0.0000257	−0.061	0.648371	0.0000258	−0.081
5	±15	0.834385	0.0000194	0.825947	0.0000215	−1.01	0.833930	0.0000196	−0.055	0.833633	0.0000196	−0.090
5	±18	0.937041	0.0000126	0.930434	0.0000148	−0.71	0.936679	0.0000127	−0.0387	0.936382	0.0000134	−0.070
5	±20	0.970678	0.0000082	0.966003	0.0000089	−0.48	0.970423	0.0000084	−0.0263	0.970185	0.0000081	−0.051
10	±0.1	0.010149	0.0000197	0.010112	0.0000173	−0.364	0.010156	0.0000207	0.061	0.010131	0.0000195	−0.181
10	±1	0.021190	0.0000183	0.020679	0.0000174	−2.41	0.021089	0.0000179	−0.48	0.021121	0.0000188	−0.323
10	±2	0.050831	0.0000168	0.048499	0.0000163	−4.6	0.050296	0.0000165	−1.05	0.050567	0.0000175	−0.52
10	±3	0.121026	0.0000199	0.113283	0.0000192	−6.4	0.119196	0.0000197	−1.51	0.120238	0.0000202	−0.65
10	±4	0.263047	0.0000241	0.243089	0.0000242	−7.6	0.258321	0.0000266	−1.80	0.261151	0.0000253	−0.72
10	±5	0.476111	0.0000273	0.438916	0.0000257	−7.8	0.467477	0.0000263	−1.81	0.472803	0.0000266	−0.69
***10***	±***5.105***	***0.500474***	***0.0000285***	***0.461588***	***0.0000261***	−***7.8***	***0.491473***	***0.0000273***	−***1.80***	***0.497048***	***0.0000272***	−***0.68***
10	±6	0.699060	0.0000248	0.650532	0.0000258	−6.9	0.688201	0.0000260	−1.55	0.695079	0.0000249	−0.57
10	±7	0.863440	0.0000182	0.818415	0.0000226	−5.2	0.853942	0.0000174	−1.10	0.860101	0.0000185	−0.387
10	±8	0.951512	0.0000122	0.920845	0.0000144	−3.22	0.945578	0.0000118	−0.62	0.949505	0.0000130	−0.211
10	±9	0.986569	0.0000074	0.970766	0.0000092	−1.60	0.983861	0.0000075	−0.274	0.985700	0.0000074	−0.088
10	±10	0.997100	0.0000031	0.990752	0.0000056	−0.64	0.996179	0.0000036	−0.092	0.996820	0.0000030	−0.0280
10	±11	0.999511	0.0000013	0.997471	0.0000029	−0.204	0.999274	0.0000016	−0.0237	0.999444	0.0000014	−0.0067
15	±0.1	0.010232	0.0000204	0.010189	0.0000210	−0.425	0.010234	0.0000209	0.0204	0.010241	0.0000200	0.089
15	±0.5	0.014693	0.0000236	0.014335	0.0000202	−2.44	0.014493	0.0000249	−1.37	0.014655	0.0000240	−0.260
15	±1	0.025057	0.0000225	0.023771	0.0000186	−5.1	0.024298	0.0000241	−3.03	0.024887	0.0000236	−0.68
15	±2.5	0.109047	0.0000212	0.097138	0.0000185	−10.9	0.102549	0.0000212	−6.0	0.107459	0.0000213	−1.46
15	±3	0.172328	0.0000245	0.151567	0.0000236	−12.0	0.161414	0.0000251	−6.3	0.169650	0.0000236	−1.55
15	±4	0.378353	0.0000288	0.330283	0.0000270	−12.7	0.355148	0.0000332	−6.1	0.372717	0.0000285	−1.49
***15***	±***4.46***	***0.501373***	***0.0000279***	***0.439974***	***0.0000313***	−***12.2***	***0.473072***	***0.0000321***	−***5.6***	***0.494564***	***0.0000297***	−***1.36***
15	±5	0.648423	0.0000269	0.576399	0.0000266	−11.1	0.617166	0.0000290	−4.82	0.641028	0.0000287	−1.14
15	±6	0.862557	0.0000198	0.795806	0.0000211	−7.7	0.837265	0.0000196	−2.93	0.856850	0.0000201	−0.66
15	±7	0.964243	0.0000093	0.925221	0.0000144	−4.05	0.951910	0.0000112	−1.28	0.961648	0.0000098	−0.269
15	±8	0.993893	0.0000043	0.978816	0.0000094	−1.52	0.990178	0.0000054	−0.374	0.993181	0.0000042	−0.072
15	±9	0.999318	0.0000015	0.995252	0.0000039	−0.407	0.998607	0.0000019	−0.071	0.999198	0.0000017	−0.0120
15	±10	0.999951	0.0000004	0.999142	0.0000017	−0.081	0.999862	0.0000007	−0.0089	0.999938	0.0000005	−0.00127
20	±0.1	0.010257	0.0000259	0.010229	0.0000248	−0.271	0.010214	0.0000243	−0.414	0.010266	0.0000277	0.086
20	±0.5	0.015400	0.0000243	0.014873	0.0000258	−3.42	0.014907	0.0000265	−3.20	0.015318	0.0000246	−0.53
20	±1	0.027246	0.0000237	0.025271	0.0000256	−7.2	0.025561	0.0000213	−6.2	0.026916	0.0000225	−1.21
20	±2.5	0.126609	0.0000249	0.108344	0.0000227	−14.4	0.113053	0.0000241	−10.7	0.123707	0.0000263	−2.29
20	±3	0.201578	0.0000265	0.170323	0.0000230	−15.5	0.179506	0.0000248	−10.9	0.196833	0.0000283	−2.35
20	±4	0.438591	0.0000283	0.371161	0.0000267	−15.4	0.395957	0.0000273	−9.7	0.429526	0.0000288	−2.07
***20***	±***4.21***	***0.499301***	***0.0000272***	***0.424653***	***0.0000266***	−***15.0***	***0.453296***	***0.0000268***	−***9.2***	***0.489576***	***0.0000279***	−***1.95***
20	±5	0.723374	0.0000244	0.634711	0.0000265	−12.3	0.674325	0.0000297	−6.8	0.713341	0.0000245	−1.39
20	±6	0.914465	0.0000163	0.847119	0.0000198	−7.4	0.883167	0.0000185	−3.42	0.908494	0.0000175	−0.65
20	±7	0.984191	0.0000070	0.953952	0.0000127	−3.07	0.973158	0.0000087	−1.12	0.982301	0.0000079	−0.192
20	±8	0.998284	0.0000021	0.989758	0.0000053	−0.85	0.996079	0.0000036	−0.221	0.997959	0.0000025	−0.0325
20	±9	0.999891	0.0000006	0.998267	0.0000024	−0.162	0.999634	0.0000011	−0.0258	0.999861	0.0000007	−0.00309
20	±10	0.999996	0.0000001	0.999771	0.0000010	−0.0225	0.999978	0.0000002	−0.00180	0.999994	0.0000001	−0.00017

**Table 5:** Power of Test (Ω) values for four single extreme outlier discordancy tests as a function of *ε*.

*n*	*ε*	Discordancy tests										
		N2		N8			N14			N15		
		${\overset{-}{x}}_{\text{N2}}$	(*u*_99_)_N2_	${\overset{-}{x}}_{\text{N8}}$	(*u*_99_)_N8_	$\Delta {\overset{-}{x}}_{\text{N8}}$	${\overset{-}{x}}_{\text{N14}}$	(*u*_99_)_N14_	$\Delta {\overset{-}{x}}_{\text{N14}}$	${\overset{-}{x}}_{\text{N15}}$	(*u*_99_)_N15_	$\Delta {\overset{-}{x}}_{\text{N15}}$
5	±1.1	0.020850	0.0000310	0.020803	0.0000314	−0.227	0.020844	0.0000307	−0.0313	0.020869	0.0000314	0.087
5	±3	0.064711	0.0000419	0.064385	0.0000419	−0.50	0.064691	0.0000425	−0.0304	0.064743	0.0000412	0.050
5	±5	0.154521	0.0000496	0.153274	0.0000481	−0.81	0.154449	0.0000483	−0.046	0.154511	0.0000480	−0.0066
5	±7	0.258413	0.0000570	0.256108	0.0000574	−0.89	0.258284	0.0000555	−0.050	0.258324	0.0000568	−0.0344
5	±10	0.397926	0.0000695	0.394635	0.0000687	−0.83	0.397744	0.0000695	−0.046	0.397724	0.0000691	−0.051
***5***	±***12.9***	***0.50175***	***0.0000610***	***0.49817***	***0.0000609***	−***0.71***	***0.50155***	***0.0000594***	−***0.0389***	***0.50151***	***0.0000620***	−***0.047***
5	±15	0.560321	0.0000690	0.556782	0.0000652	−0.63	0.560129	0.0000682	−0.0341	0.560080	0.0000690	−0.0429
5	±20	0.660616	0.0000600	0.657405	0.0000600	−0.49	0.660442	0.0000600	−0.0264	0.660386	0.0000588	−0.0347
5	±30	0.771476	0.0000629	0.769007	0.0000655	−0.320	0.771337	0.0000637	−0.0181	0.771296	0.0000644	−0.0233
5	±40	0.822288	0.0000430	0.820357	0.0000442	−0.235	0.822181	0.0000431	−0.0130	0.822138	0.0000428	−0.0182
5	±60	0.881517	0.0000389	0.880180	0.0000385	−0.152	0.881443	0.0000392	−0.0084	0.881408	0.0000394	−0.0124
5	±80	0.911274	0.0000321	0.910263	0.0000327	−0.111	0.911218	0.0000324	−0.0061	0.911195	0.0000311	−0.0087
5	±120	0.941005	0.0000307	0.940328	0.0000307	−0.072	0.940970	0.0000306	−0.00371	0.940951	0.0000306	−0.0058
5	±160	0.955830	0.0000281	0.955330	0.0000269	−0.052	0.955801	0.0000285	−0.00302	0.955792	0.0000281	−0.00395
5	±200	0.964715	0.0000244	0.964312	0.0000248	−0.0417	0.964693	0.0000241	−0.00226	0.964681	0.0000242	−0.00345
10	±1.1	0.023252	0.0000400	0.023052	0.0000335	−0.86	0.023228	0.0000400	−0.106	0.023210	0.0000386	−0.181
10	±3	0.230007	0.0000688	0.215369	0.0000615	−6.4	0.226613	0.0000671	−1.48	0.228723	0.0000691	−0.56
10	±5	0.466178	0.0000675	0.444482	0.0000696	−4.7	0.461346	0.0000693	−1.037	0.464412	0.0000674	−0.379
***10***	±***5.4***	***0.501202***	***0.0000668***	***0.479497***	***0.0000682***	−***4.33***	***0.496385***	***0.0000629***	−***0.96***	***0.499458***	***0.0000674***	−***0.348***
10	±10	0.609736	0.0000680	0.589504	0.0000642	−3.32	0.605299	0.0000668	−0.73	0.608161	0.0000726	−0.258
10	±10	0.729058	0.0000551	0.712878	0.0000551	−2.22	0.725561	0.0000548	−0.48	0.727835	0.0000575	−0.168
10	±15	0.824592	0.0000517	0.813173	0.0000531	−1.38	0.822123	0.0000498	−0.299	0.823755	0.0000530	−0.101
10	±20	0.872018	0.0000545	0.863336	0.0000533	−1.00	0.870142	0.0000543	−0.215	0.871397	0.0000552	−0.071
10	±30	0.918594	0.0000550	0.912810	0.0000579	−0.63	0.917335	0.0000538	−0.137	0.918194	0.0000540	−0.0435
10	±40	0.934126	0.0000272	0.929776	0.0000294	−0.47	0.933203	0.0000279	−0.099	0.933789	0.0000276	−0.0360
10	±50	0.947611	0.0000234	0.944143	0.0000241	−0.366	0.946875	0.0000242	−0.078	0.947342	0.0000238	−0.0284
10	±100	0.974142	0.0000183	0.972421	0.0000184	−0.177	0.973777	0.0000185	−0.0374	0.974010	0.0000184	−0.0136
10	±150	0.982843	0.0000161	0.981700	0.0000168	−0.116	0.982600	0.0000166	−0.0247	0.982756	0.0000161	−0.0089
10	±200	0.987171	0.0000142	0.986318	0.0000144	−0.086	0.986991	0.0000138	−0.0183	0.987105	0.0000143	−0.0067
15	±1.1	0.024941	0.0000507	0.024516	0.0000434	−1.70	0.024714	0.0000454	−0.91	0.024907	0.0000523	−0.137
15	±3	0.315624	0.0000816	0.285482	0.0000707	−9.6	0.301908	0.0000791	−4.35	0.312616	0.0000768	−0.95
***15***	±***4.4***	***0.505574***	***0.0000745***	***0.470626***	***0.0000677***	−***6.9***	***0.490624***	***0.0000697***	−***2.96***	***0.502393***	***0.0000705***	−***0.63***
15	±5	0.563420	0.0000689	0.529313	0.0000721	−6.1	0.549047	0.0000748	−2.55	0.560407	0.0000698	−0.53
15	±7	0.692305	0.0000627	0.663682	0.0000610	−4.13	0.680599	0.0000638	−1.69	0.689963	0.0000629	−0.338
15	±10	0.791754	0.0000591	0.770289	0.0000590	−2.71	0.783169	0.0000580	−1.08	0.790166	0.0000564	−0.201
15	±15	0.867777	0.0000506	0.853207	0.0000541	−1.68	0.862030	0.0000516	−0.66	0.866870	0.0000525	−0.105
15	±20	0.904654	0.0000486	0.893754	0.0000511	−1.20	0.900431	0.0000428	−0.47	0.904100	0.0000549	−0.061
15	±30	0.940391	0.0000448	0.933225	0.0000533	−0.76	0.937686	0.0000478	−0.288	0.940194	0.0000497	−0.0209
15	±40	0.950189	0.0000256	0.944782	0.0000263	−0.57	0.948017	0.0000256	−0.229	0.949688	0.0000258	−0.053
15	±60	0.967191	0.0000212	0.963613	0.0000224	−0.370	0.965751	0.0000218	−0.149	0.966859	0.0000207	−0.0343
15	±80	0.975546	0.0000199	0.972878	0.0000213	−0.274	0.974475	0.0000198	−0.110	0.975299	0.0000200	−0.0253
15	±140	0.986132	0.0000140	0.984621	0.0000148	−0.153	0.985524	0.0000143	−0.062	0.985992	0.0000137	−0.0142
15	±200	0.990334	0.0000113	0.989278	0.0000127	−0.107	0.989908	0.0000116	−0.0429	0.990235	0.0000116	−0.0099
20	±1.1	0.025959	0.0000600	0.025296	0.0000570	−2.551	0.025396	0.0000534	−2.17	0.025877	0.0000610	−0.313
20	±3	0.362436	0.0000753	0.321879	0.0000782	−11.2	0.338086	0.0000708	−6.7	0.357843	0.0000754	−1.27
***20***	±***4***	***0.509029***	***0.0000677***	***0.465277***	***0.0000661***	−***8.6***	***0.484093***	***0.0000691***	−***4.9***	***0.504506***	***0.0000649***	−***0.89***
20	±5	0.609022	0.0000669	0.567957	0.0000706	−6.7	0.586331	0.0000707	−3.73	0.605042	0.0000639	−0.65
20	±7	0.728947	0.0000692	0.695836	0.0000693	−4.5	0.711240	0.0000640	−2.43	0.726084	0.0000696	−0.393
20	±10	0.818758	0.0000462	0.794479	0.0000533	−2.97	0.806135	0.0000486	−1.54	0.816978	0.0000476	−0.217
20	±15	0.886139	0.0000424	0.869861	0.0000430	−1.84	0.877932	0.0000468	−0.93	0.885261	0.0000465	−0.099
20	±20	0.918508	0.0000399	0.906382	0.0000446	−1.32	0.912559	0.0000407	−0.65	0.918085	0.0000427	−0.046
20	±30	0.949667	0.0000432	0.941715	0.0000507	−0.84	0.945966	0.0000481	−0.390	0.949717	0.0000506	0.0053
20	±40	0.956868	0.0000229	0.950924	0.0000258	−0.62	0.953687	0.0000219	−0.332	0.956225	0.0000226	−0.067
20	±60	0.971612	0.0000209	0.967685	0.0000218	−0.404	0.969514	0.0000209	−0.216	0.971191	0.0000210	−0.0434
20	±80	0.978856	0.0000170	0.975928	0.0000185	−0.299	0.977289	0.0000179	−0.160	0.978541	0.0000170	−0.0322
20	±140	0.988015	0.0000127	0.986356	0.0000127	−0.168	0.987127	0.0000139	−0.090	0.987837	0.0000128	−0.0181
20	±200	0.991646	0.0000107	0.990487	0.0000113	−0.117	0.991025	0.0000109	−0.063	0.991520	0.0000113	−0.0126

**Table 6:** Test Performance Criterion P5 (π_*D*∣*C*_) values for four single extreme outlier discordancy tests as a function of *ε*.

*n*	*ε*	Discordancy tests										
		N2		N8			N14			N15		
		${\overset{-}{x}}_{\text{N2}}$	(*u*_99_)_N2_	${\overset{-}{x}}_{\text{N8}}$	(*u*_99_)_N8_	$\Delta {\overset{-}{x}}_{\text{N8}}$	${\overset{-}{x}}_{\text{N14}}$	(*u*_99_)_N14_	$\Delta {\overset{-}{x}}_{\text{N14}}$	${\overset{-}{x}}_{\text{N15}}$	(*u*_99_)_N15_	$\Delta {\overset{-}{x}}_{\text{N15}}$
5	±1.1	0.011106	0.0000274	0.011080	0.0000273	−0.237	0.011101	0.0000274	−0.0425	0.011115	0.0000280	0.076
5	±3	0.056787	0.0000393	0.056474	0.0000387	−0.55	0.056768	0.0000397	−0.0321	0.056810	0.0000382	0.0408
5	±5	0.146913	0.0000462	0.145680	0.0000452	−0.84	0.146842	0.0000447	−0.048	0.146893	0.0000452	−0.0131
5	±7	0.250890	0.0000523	0.248598	0.0000537	−0.91	0.250762	0.0000512	−0.051	0.250790	0.0000524	−0.0397
5	±10	0.390462	0.0000624	0.387183	0.0000608	−0.84	0.390280	0.0000627	−0.047	0.390249	0.0000624	−0.055
***5***	±***13.1***	***0.50043***	***0.0000544***	***0.49686***	***0.0000559***	−***0.71***	***0.50023***	***0.0000544***	−***0.0406***	***0.50018***	***0.0000553***	−***0.050***
5	±15	0.552883	0.0000554	0.549351	0.0000535	−0.64	0.552692	0.0000547	−0.0346	0.552630	0.0000551	−0.046
5	±20	0.653179	0.0000491	0.649982	0.0000501	−0.49	0.653009	0.0000491	−0.0262	0.652941	0.0000480	−0.0365
5	±30	0.764071	0.0000476	0.761610	0.0000464	−0.322	0.763935	0.0000480	−0.0178	0.763882	0.0000487	−0.0248
5	±40	0.822288	0.0000430	0.820357	0.0000442	−0.235	0.822181	0.0000431	−0.0130	0.822138	0.0000428	−0.0182
5	±60	0.881517	0.0000389	0.880180	0.0000385	−0.152	0.881443	0.0000392	−0.0084	0.881408	0.0000394	−0.0124
5	±80	0.911274	0.0000321	0.910263	0.0000327	−0.111	0.911218	0.0000324	−0.0061	0.911195	0.0000311	−0.0087
5	±120	0.941005	0.0000307	0.940328	0.0000307	−0.072	0.940970	0.0000306	−0.00371	0.940951	0.0000306	−0.0058
5	±160	0.955830	0.0000281	0.955330	0.0000269	−0.052	0.955801	0.0000285	−0.00302	0.955792	0.0000281	−0.00395
5	±200	0.964715	0.0000244	0.964312	0.0000248	−0.0417	0.964693	0.0000241	−0.00226	0.964681	0.0000242	−0.00345
10	±1.1	0.013593	0.0000385	0.013418	0.0000340	−1.29	0.013562	0.0000384	−0.227	0.013564	0.0000378	−0.209
10	±3	0.222304	0.0000659	0.207623	0.0000591	−6.6	0.218930	0.0000661	−1.52	0.220981	0.0000656	−0.60
10	±5	0.458753	0.0000661	0.437008	0.0000668	−4.7	0.453946	0.0000689	−1.05	0.456939	0.0000667	−0.395
***10***	±***5.41***	***0.499554***	***0.0000668***	***0.477805***	***0.0000682***	−***4.35***	***0.494764***	***0.0000629***	−***0.96***	***0.49776***	***0.0000674***	−***0.359***
10	±7	0.602388	0.0000651	0.582111	0.0000625	−3.37	0.597979	0.0000649	−0.73	0.600766	0.0000684	−0.269
10	±10	0.721760	0.0000501	0.705530	0.0000527	−2.25	0.718290	0.0000492	−0.48	0.720482	0.0000517	−0.177
10	±15	0.817312	0.0000418	0.805848	0.0000426	−1.40	0.814868	0.0000429	−0.299	0.816420	0.0000412	−0.109
10	±20	0.864739	0.0000433	0.856024	0.0000408	−1.01	0.862890	0.0000426	−0.214	0.864058	0.0000439	−0.079
10	±30	0.911335	0.0000299	0.905514	0.0000319	−0.64	0.910096	0.0000294	−0.136	0.910883	0.0000309	−0.050
10	±40	0.934126	0.0000272	0.929776	0.0000294	−0.47	0.933203	0.0000279	−0.099	0.933789	0.0000276	−0.0360
10	±50	0.947611	0.0000234	0.944143	0.0000241	−0.366	0.946875	0.0000242	−0.078	0.947342	0.0000238	−0.0284
10	±100	0.974142	0.0000183	0.972421	0.0000184	−0.177	0.973777	0.0000185	−0.0374	0.974010	0.0000184	−0.0136
10	±150	0.982843	0.0000161	0.981700	0.0000168	−0.116	0.982600	0.0000166	−0.0247	0.982756	0.0000161	−0.0089
10	±200	0.987171	0.0000142	0.986318	0.0000144	−0.086	0.986991	0.0000138	−0.0183	0.987105	0.0000143	−0.0067
15	±1.1	0.015232	0.0000509	0.014804	0.0000414	−2.81	0.014971	0.0000442	−1.71	0.015180	0.0000518	−0.340
15	±3	0.307670	0.0000774	0.277454	0.0000694	−9.8	0.293796	0.0000758	−4.5	0.304363	0.0000734	−1.08
***15***	±***4.5***	***0.508306***	***0.0000693***	***0.473352***	***0.0000627***	−***6.9***	***0.493247***	***0.0000686***	−***2.96***	***0.504762***	***0.0000674***	−***0.70***
15	±5	0.555712	0.0000644	0.521524	0.0000708	−6.2	0.541147	0.0000721	−2.62	0.552297	0.0000664	−0.61
15	±7	0.684663	0.0000590	0.655963	0.0000589	−4.19	0.672765	0.0000603	−1.74	0.681886	0.0000602	−0.406
15	±10	0.784156	0.0000518	0.762607	0.0000574	−2.75	0.775363	0.0000548	−1.12	0.782114	0.0000522	−0.260
15	±15	0.860198	0.0000416	0.845545	0.0000444	−1.70	0.854249	0.0000423	−0.69	0.858831	0.0000422	−0.159
15	±20	0.897091	0.0000348	0.886105	0.0000350	−1.22	0.892656	0.0000352	−0.49	0.896063	0.0000350	−0.115
15	±30	0.932834	0.0000271	0.925572	0.0000285	−0.78	0.929908	0.0000291	−0.314	0.932159	0.0000271	−0.072
15	±40	0.950189	0.0000256	0.944782	0.0000263	−0.57	0.948017	0.0000256	−0.228	0.949688	0.0000258	−0.053
15	±60	0.967191	0.0000212	0.963613	0.0000224	−0.370	0.965751	0.0000218	−0.149	0.966859	0.0000207	−0.0343
15	±80	0.975546	0.0000199	0.972878	0.0000213	−0.274	0.974475	0.0000198	−0.110	0.975299	0.0000200	−0.0253
15	±140	0.986132	0.0000140	0.984621	0.0000148	−0.153	0.985524	0.0000143	−0.062	0.985992	0.0000137	−0.0142
15	±200	0.990334	0.0000113	0.989278	0.0000127	−0.107	0.989908	0.0000116	−0.0429	0.990235	0.0000116	−0.0099
20	±1.1	0.016228	0.0000577	0.015531	0.0000546	−4.29	0.015647	0.0000516	−3.58	0.016119	0.0000580	−0.67
20	±3	0.354224	0.0000726	0.313612	0.0000769	−11.5	0.329474	0.0000698	−7.0	0.349056	0.0000732	−1.46
***20***	±***4***	***0.500954***	***0.0000639***	***0.457154***	***0.0000653***	−***8.7***	***0.475548***	***0.0000651***	−***5.1***	***0.495727***	***0.0000620***	−***1.04***
20	±5	0.601015	0.0000617	0.559904	0.0000661	−6.8	0.577809	0.0000665	−3.86	0.596262	0.0000581	−0.79
20	±7	0.721004	0.0000628	0.687842	0.0000622	−4.6	0.702741	0.0000577	−2.53	0.717289	0.0000637	−0.52
20	±10	0.810844	0.0000448	0.786525	0.0000468	−3.0	0.797652	0.0000469	−1.63	0.808171	0.0000433	−0.330
20	±15	0.878244	0.0000370	0.861918	0.0000398	−1.86	0.869456	0.0000419	−1.00	0.876461	0.0000382	−0.203
20	±20	0.910617	0.0000332	0.898443	0.0000353	−1.34	0.904086	0.0000333	−0.72	0.909299	0.0000325	−0.145
20	±30	0.941779	0.0000246	0.933781	0.0000270	−0.85	0.937486	0.0000248	−0.46	0.940911	0.0000255	−0.092
20	±40	0.956868	0.0000229	0.950924	0.0000258	−0.62	0.953687	0.0000219	−0.332	0.956225	0.0000226	−0.067
20	±60	0.971612	0.0000209	0.967685	0.0000218	−0.404	0.969514	0.0000209	−0.216	0.971191	0.0000210	−0.0434
20	±80	0.978856	0.0000170	0.975928	0.0000185	−0.299	0.977289	0.0000179	−0.160	0.978541	0.0000170	−0.0322
20	±140	0.988015	0.0000127	0.986356	0.0000127	−0.168	0.987127	0.0000139	−0.090	0.987837	0.0000128	−0.0181
20	±200	0.991646	0.0000107	0.990487	0.0000113	−0.117	0.991025	0.0000109	−0.063	0.991520	0.0000113	−0.0126
